# On the Wavelength-Dependent Photochemistry of the Atmospheric Molecule CF_3_COCl

**DOI:** 10.1021/acsearthspacechem.3c00196

**Published:** 2023-10-16

**Authors:** Jiří Janoš, Ivo S. Vinklárek, Jozef Rakovský, Deb Pratim Mukhopadhyay, Basile F. E. Curchod, Michal Fárník, Petr Slavíček

**Affiliations:** † Department of Physical Chemistry, University of Chemistry and Technology, Technická 5, Prague 6 166 28, Czech Republic; ‡ Department of Dynamics of Molecules and Clusters, J. Heyrovský Institute of Physical Chemistry, v.v.i., The Czech Academy of Sciences, Dolejškova 2155/3, 182 23 Prague, Czech Republic; § Centre for Computational Chemistry, School of Chemistry, University of Bristol, Bristol BS8 1TS, U.K.

**Keywords:** computational photochemistry, conical intersections, excited states, molecular beams, photodissociation, velocity map imaging, photolysis

## Abstract

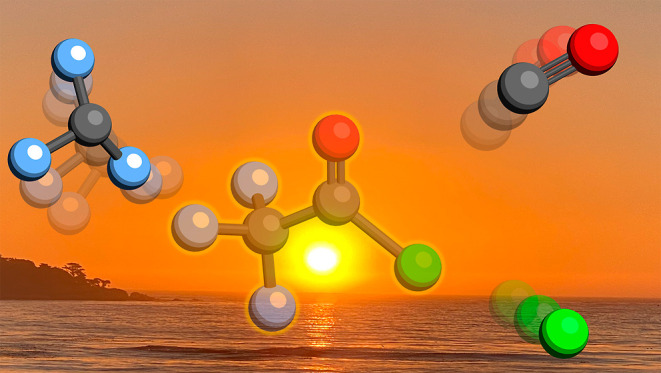

The wavelength control of photochemistry usually results from ultrafast dynamics following the excitation of different electronic states. Here, we investigate the CF_3_COCl molecule, exhibiting wavelength-dependent photochemistry both via (i) depositing increasing internal energy into a single state and (ii) populating different electronic states. We reveal the mechanism behind the photon-energy dependence by combining nonadiabatic *ab initio* molecular dynamics techniques with the velocity map imaging experiment. We describe a consecutive mechanism of photodissociation where an immediate release of Cl taking place in an excited electronic state is followed by a slower ground-state dissociation of the CO fragment. The CO release is subject to an activation barrier and is controlled by excess internal energy via the excitation wavelength. Therefore, a selective release of CO along with Cl can be achieved. The mechanism is fully supported by both the measured kinetic energy distributions and anisotropies of the angular distributions. Interestingly, the kinetic energy of the released Cl atom is sensitively modified by accounting for spin–orbit coupling. Given the atmospheric importance of CF_3_COCl, we discuss the consequences of our findings for atmospheric photochemistry.

## 1 Introduction

Molecules bearing a carbonyl group constitute an important family of transient volatile organic compounds in the troposphere. Their photochemistry is typically governed by Norrish-type cleavage, following a fast intersystem crossing upon excitation by sunlight. Their reactivity following photoexcitation is, however, significantly altered with the addition of halogens, opening new deactivation and photolysis channels. An example of such a molecule is CF_3_COCl.

CF_3_COCl is formed from tropospheric photo-oxidative degradation of hydrochlorofluorocarbons and brominated species,^1,2^ which are often used as a replacement for ozone-depleting chlorofluorocarbon compounds. The presence of these molecules in our atmosphere has increased in the last decades, and understanding their possible sink through photolysis and photoproducts is of first importance.^3−5^ Various experimental techniques including ultraviolet (UV) absorption,^5−7^ infrared (IR)^5,6,8^ or vibrational spectroscopy,^9^ and electron diffraction^10^ supported by theoretical calculations^1,11^ were applied to investigate the photochemistry of CF_3_COCl and its role in atmospheric chemistry. The fully halogenated carbonyl compounds, including CF_3_COCl, follow either degradation by UV fragmentation or wet deposition and hydrolysis. Both channels have comparable importance in the troposphere, while photolysis becomes dominant at higher altitudes.^2,3,5^ Mechanism of the wavelength-dependent photolysis of compounds like CF_3_COCl is therefore of key importance for understanding their impact on the composition of our atmosphere.

**Figure 1:**
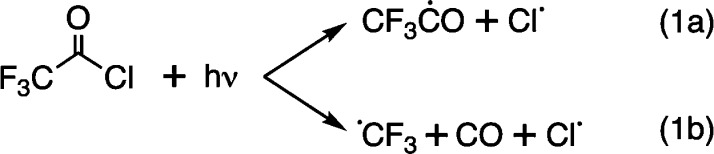
Two major photochemical pathways of CF_3_COCl.

The photochemistry of CF_3_COCl is rather rich and different from the usual carbonyl photochemistry. The UV absorption spectrum of CF_3_COCl exhibits two bands: the first is centered at ∼250 nm and the second is found at shorter wavelengths with a red edge reaching up to ∼215 nm. As further detailed below, the first absorption band corresponds to a single electronically excited state, whereas the second band consists of more excited states. Previous experimental studies^2,5^ reported a strong dependence of fragmentation pattern on the excitation energy between 193 and 280 nm with two major pathways (see Figure 1). While the single-bond C–Cl cleavage is a preferential pathway for photons with wavelengths above 260 nm, the three-body fragmentation prevails in the high-energy part of the UV absorption spectrum.^5^ Two additional minor channels were also reported in early studies corresponding to the direct C–C bond cleavage and generation of CF_3_Cl species.^7^ However, an upper limit of their relative contribution was later estimated below <0.004.^5^ From a computational perspective, the photochemistry of CF_3_COCl upon photoexcitation at a single wavelength (254 nm) was recently simulated by Hao et al.^11^

In the present work, we show how the photochemical dynamics of such molecules can be fully understood by combining the velocity map imaging (VMI) technique with *ab initio* excited-state dynamics simulations. The photochemistry of CF_3_COCl challenges the (common) assumption that the photochemical reactivity usually takes place in the lowest singlet or triplet state.^12^ This assumption is consistent with Kasha–Vavilov’s rule, noting that the fluorescence usually takes place from the lowest singlet state and is, as such, independent of the incident wavelength.^13^ Recently, different works have focused on photochemical processes that deviate from this assumption, particularly when taking place in the gas phase. For example, photoproducts can be formed in the ground electronic state following nonradiative decay thanks to athermal (nonstatistical) processes,^14,15^ and some photochemical reactions clearly exhibit a wavelength-dependent reactivity, e.g. photochemistry of heterocycles,^16^ photolysis of glyoxal and volatile organic compounds,^17,18^ photoinitiating reactions,^19^ or chemistry of phytochromophores.^20^ The wavelength-dependent formation of photoproducts for these examples is, however, easily explained by the distinct electronic states that can be populated using different excitation wavelengths, ultimately leading to diverse reaction pathways. In atmospheric chemistry, wavelength-dependent photolysis quantum yields are far from uncommon. This wavelength dependence can be either interstate—distinct photochemistry due to different excited electronic states—or intrastate—distinct photochemistry within the same electronic state due to a different internal energy. CF_3_COCl is exciting in this sense as it exhibits both types of wavelength-dependent photochemistry and as such constitutes a perfect molecule to test the combination of VMI and excited-state molecular dynamics to unravel the mechanistic details of each nonradiative pathway.

In the following, we unravel the different mechanisms causing the wavelength dependence of the photochemistry of CF_3_COCl when promoted into (i) its first excited electronic state with different internal energies or (ii) its second/third excited electronic states. Our work provides a detailed molecular-level understanding of the possible photodegradation channels of CF_3_COCl in the atmosphere, highlighting more specifically the importance of the ground-state dynamics following nonradiative decay in the formation of photoproducts and the influence that the (weak) spin–orbit coupling of the Cl atom can have on the dynamics.

## 2 Methods

### 2.1 Velocity Map Imaging

The experimental data were acquired on our apparatus for imaging (AIM) described first in ref (21), which is based on a VMI technique.^22,23^ The general idea of the VMI is a projection of an expanding sphere of photofragments after photodissociation onto a position-sensitive detector. The position where the photofragment lands on the detector provides information about the fragment velocity vector, i.e., its speed and direction of flight with respect to the laser polarization plane. Thus, by reconstructing the recorded images by a mathematical procedure, the full 3D information about the fragment velocity can be recovered, which in turn provides detailed information about the photodissociation dynamics.

The experimental procedures were similar to the ones described recently for the photodissociation of methyl chloride^24^ and higher chloroalkanes.^25^ The sample vapor of CF_3_COCl (abcr GmbH) was premixed in a stainless-steel gas container with helium buffer gas into 1% mixture (1.3 bar) and connected through inlet line to the pulsed nozzle (General Valve) in the source chamber (background vacuum 1 × 10^–7^ mbar). The nozzle opens at a repetition rate of 10 Hz, and the supersonic expansion is skimmed to the next chamber to generate a molecular beam. The second chamber (3 × 10^–8^ mbar) is equipped with the VMI spectrometer with perpendicular geometry according to the design of Eppink and Parker.^22^ In the interaction region of the spectrometer, the molecular beam is crossed with laser beams for excitation and fragment ionization. First, molecules were photodissociated by a short laser pulse (5 ns) with a tunable wavelength in the range of 193 nm −280 nm. We recorded velocity map images for three different fragments separately: Cl(^2^P_3/2_), Cl*(^2^P_1/2_) (^35^Cl isotopes), and CO(ν = 0); the fragments were ionized by the second pulse tuned to the wavelength for resonance-enhanced multiphoton ionization (REMPI) at 235.31, 235.17, and 230.046 nm, respectively. For the CO fragment, several low-lying *J* states can be excited within our laser line-width. Three different lasers were applied during the experiments: (a) excimer ArF laser (Coherent ExciStar, 0.3 mJ/pulse) lasing at 193 nm; (b) UV optical parametric oscillators (UV OPO) Nd/YAG laser (NT 230, Ekspla, line-width ∼4 cm^–1^) applied for the photodissociation at 235, 254, and 280 nm, and for REMPI at single-color experiments at the wavelengths outlined above; and (c) dye laser (PULSARE, fine adjustment, rhodamine B dye, line-width ∼0.5 cm^–1^) applied for REMPI at two-color experiments. The polarization of all lasers is set in parallel with the plane of the detector. The generated ions are extracted by the field between the repeller and the extractor in the direction of the detector, which is composed of a microchannel plate with a phosphor screen (P43) and a CCD camera (Unibrain). The ion images are recorded in ion counting mode (self-made LabView program) and later processed by the standard procedure of direct inverse Abel transformation to gain VMI images (PyAbel Python package).^26^ Kinetic energy distributions (KEDs) and angular distributions are evaluated from the images by standard integration procedures.^23^ Energy calibration was performed by a single-laser photodissociation experiment of HBr at 243.1 nm. The time synchronization of the experiments is driven by two delay generators (BNC 575).

### 2.2 Computational Modeling

#### 2.2.1 Electronic Structure

The potential energy curves along the dissociation coordinates were investigated with the complete active space self-consistent field (CASSCF) method. As the processes studied in this work are very fast, we did not study the topology of the triplet manifold of electronic states. Six singlet states were calculated with the state-averaging (SA) procedure. The active space consisted of 12 electrons in 9 orbitals (σ_CC_, σ_CCl_, π_CO_, n_CO_, p_Cl,y_, p_Cl,*x*_, π_CO_*, σ_CCl_*, and σ_CC_*) denoted as (12,9). Including this relatively large number of states and orbitals is important as the Cl dissociation correlates to triply degenerate electronic states. The SA2-CASSCF(8,7) scheme previously used by Hao et al.^11^ lacks the two p_Cl_ orbitals and four higher excited states required for the correct dissociation limit; see the Supporting Information. The dynamic correlation was included via the extended multistate complete active space second-order perturbation theory (XMS-CASPT2) method^27^ based on the CASSCF reference wave function.

Although applicable for the static description, the active space (12,9) is unsuitable for nonadiabatic dynamics simulations because it suffers from energy discontinuities along the propagation. The spurious behavior stems from the vibration of the C=O bond, which drives the chemically irrelevant σ_CO_ and σ_CO_* orbitals into the active space instead of the σ_CC_ and σ_CC_* orbitals necessary for dissociative dynamics. The (14,11) active space with additional σ_CO_ and σ_CO_* orbitals circumvents the problem, yet this large space is beyond our computational power. Thus, we used an alternative method of floating occupation molecular orbitals complete active space configuration interaction (FOMO-CASCI)^28,29^ that alleviates the cost of orbital optimization and makes bigger active spaces feasible. Also, the number of states included in the calculations can be decreased as no SA procedure is included in FOMO-CASCI. The broadening parameter β in the FOMO-CASCI method was set to 0.30 au as the result of variational optimization of the lowest three singlet states in the ground-state geometry. During the nonadiabatic dynamics simulations, spin-impure states appeared when the C=O bond was prolonged. Therefore, we used a Gram–Schmidt spin projection scheme for the purification of the states as proposed by Fales et al.^30^

The dynamic correlation is an essential factor during dissociation, and uncorrelated FOMO-CASCI and CASSCF methods strongly underestimate the dissociation energies and activation barriers in the *S*_1_ state. Dynamical correlation can be (approximately) included with methods such as XMS-CASPT2, but these approaches remain computationally expensive. Therefore, we applied an empirical correlation correction (ECC) to the FOMO-CASCI energies (further denoted as FOMO-CASCI/ECC) along the Cl and CO dissociation channels.^31^ The ECC has a form of a modified Morse potential, which nicely fits the difference between the XMS-CASPT2 and FOMO-CASCI energies
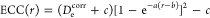
1where *r* is the bond length along which the ECC is applied, *D*_e_^corr^ is the difference between XMS-CASPT2 and FOMO-CASCI dissociation energies (this parameter was not fitted), and *a*, *b*, and *c* are the fitted parameters.

**Figure 2:**
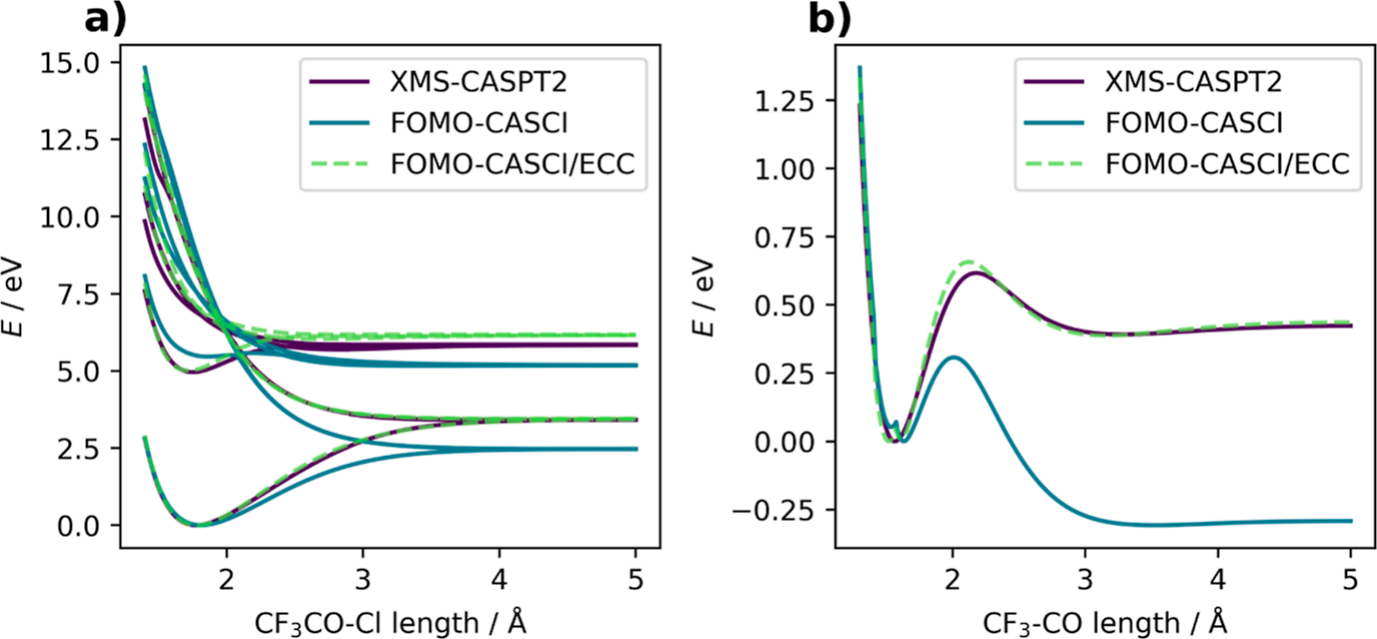
Electronic energies at the XMS-CASPT2, FOMO-CASCI, and FOMO-CASCI/ECC levels of theory along the two coordinates that were used for fitting: (a) the CF_3_CO–Cl bond and (b) the CF_3_–CO bond with the Cl atom dissociated.

The correction was applied along two dissociation coordinates: the C–Cl and C–C bonds. First, let us discuss the ECC for C–Cl. We adopted two strategies: (a) we used separate corrections for the ground state and for the excited states to achieve the best accuracy for all states (see Figure 2a). The *D*_e_^corr^ was evaluated for the lower dissociation limit, and it was used for both the ground and excited states. The correction for the excited states was fitted on the *S*_1_ state since it is the state of interest for all the pulses in the first absorption band. Although fitted on *S*_1_, it significantly improved all of the other excited states. The ground state was fitted separately as it required different parameters to achieve a satisfactory agreement with the XMS-CASPT2 calculations. Different corrections for the ground state allowed for significant improvements in the absorption spectra and a better selection of initial conditions. (b) In nonadiabatic dynamics, we applied a single ECC for all the states so that we do not affect the position of the conical intersections. Thus, the ground state was treated with the same ECC as the excited states in (a). This change from (a) makes no effective difference because all the dynamics happen in the excited states and simulations never reach the ground-state minimum. Yet, it was necessary to prevent any possible artificial energy crossings between the *S*_0_ and *S*_1_ states created by different ECCs in (a). Those artificial crossings appeared when the C=O bond prolonged and the *S*_0_ and *S*_1_ states approach 0.5 eV on the FOMO-CASCI level but cross with the ECC.

Second, ECC was applied to the C–C bond (see Figure 2b). The fit was performed with Cl dissociated (it was kept 10 Å away from the CF_3_CO fragment) since it dissociates first and CO is released from the ground state of the CF_3_CO. The CO dissociation from CF_3_COCl is subject to a high barrier and was never observed in simulations with no ECC. Therefore, we used just the ground-state PESs of the CF_3_CO fragment for the fitting. We see that ECC improves the FOMO-CASCI to the XMS-CASPT2 level and makes the CO dissociation endothermic instead of exothermic. This correction should not influence the CF_3_COCl dynamics, as it just strengthens the C–C bond, which would not dissociate before Cl anyway. All of the fitting parameters are presented in Table 1. More details about both ECCs can be found in the Supporting Information.

**Table 1:** Fitting Parameters for the ECC (eq 1)

bond	fitted state	*D*_e_^corr^/a.u.	*a*/a.u.	*b*/a.u.	*c*/a.u.
C–Cl	*S*_0_	0.03473471	0.76227003	3.05419933	0.00327769
C–Cl	*S*_1_	0.03473471	1.27833752	2.97967498	0.02708953
C–C	*S*_0_	0.02629121	1.06784164	2.66783831	0.00375042

Dissociation energies were also calculated with the coupled clusters with singles and doubles (CCSD) and CCSD with perturbative triples [CCSD(T)] methods. The ground-state frequencies for harmonic Wigner sampling were calculated with the PBE0 method. The 6-31G* basis set was applied in all calculations since the recalculation of the PES with 6-31+G* and 6-311+G** basis sets showed no significant difference. The CASSCF and XMS-CASPT2 calculations were performed in the Molpro^32^ and OpenMolcas packages,^33,34^ respectively; the PBE0, CCSD, and CCSD(T) calculations were done in the Gaussian 09 package.^35^ The FOMO-CASCI method was used in its TeraChem *ab initio* implementation.^36,37^

#### 2.2.2 Photoabsorption Cross-Section

The ground state was sampled with 10,000 geometries from the harmonic Wigner distribution using PBE0/aug-cc-pVQZ frequencies. The excitation energies and transition dipole moments were calculated at the SA6-CASSCF(12,9), XMS-CASPT2(12,9), and FOMO-CASCI(14,11) levels. The FOMO-CASCI energies were corrected with eq 1 described above using different ECC for the ground state and the excited states. The photoabsorption cross-section was calculated with the nuclear ensemble approach with a broadening parameter equal to 0.08 eV.^38^

#### 2.2.3 Nonadiabatic Dynamics

The nonadiabatic dynamics were modeled with the adiabatic Landau–Zener surface hopping (LZSH) method,^39,40^ which proved stable and efficient for this system. The LZSH method is a simple technique suitable for well-defined peaked conical intersections.^41^ This is the case of the CF_3_COCl molecule since the dissociation in the first excited states is an intersection-free process and there are well-defined conical intersections in the higher excited states. Besides that, calculations of nonadiabatic couplings required by advanced algorithms eventually failed when Cl dissociated, and the electronic states became degenerate. Thus, a nonadiabatic coupling-free method was necessary. FOMO-CASCI with the ECC described above was used for the underlying potential energy. The nonadiabatic dynamics were calculated in our in-house code ABIN.^42^

The initial conditions for the dynamics were chosen from the distribution generated for the spectrum calculation, as described above. From that distribution, we selected geometries with excitation energies corresponding to the experimental wavelengths. Only geometries with non-negligible transition dipole moments were included in the initial conditions because the kinetic energies were weighted by the transition probability when modeling the experimental signal. Corresponding momenta were generated along with the geometries at the same level.

For the KEDs, we calculated the kinetic energy of the outgoing fragments, if dissociated, and represented them by a Gaussian function weighted by the transition dipole moments in order to create a smooth distribution. The widths of the Gaussians were set so that the distribution is smooth but retained all of the important features.

## 3 Results

### 3.1 Photoabsorption Cross-Section of CF_3_COCl

**Figure 3:**
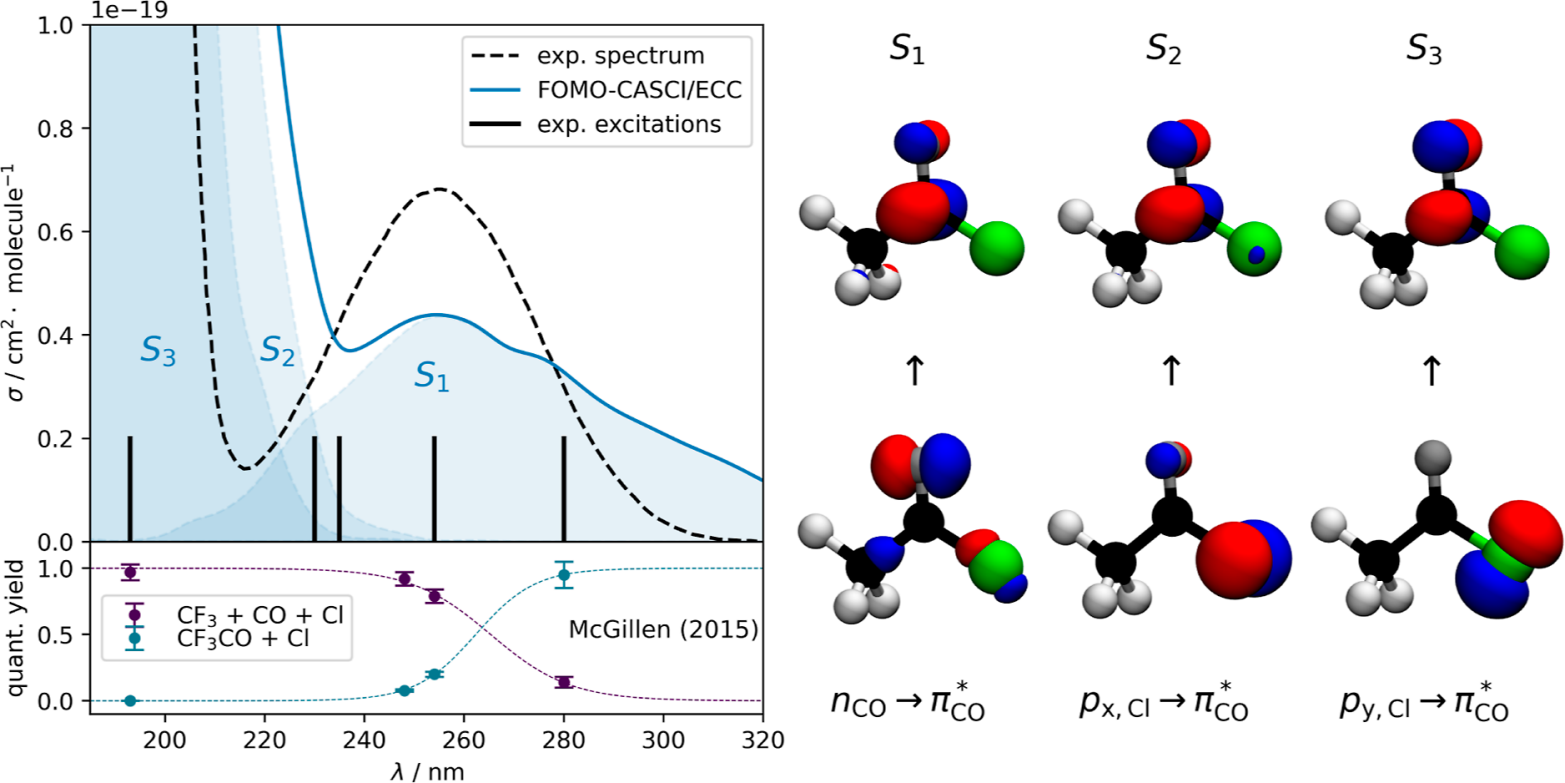
Calculated (solid line, FOMO-CASCI/ECC) and experimental (dashed line, ref (7)) photoabsorption cross-section of CF_3_COCl. The selected excitation wavelengths are depicted by black sticks. The calculated contributions from the different electronic transitions are highlighted by the shaded areas. The quantum yields in the lower left panel were adapted from ref (5). The natural orbitals (FOMO-CASCI) describing each transition are given in the right panel. The FOMO-CASCI/ECC cross-section was benchmarked against the SA6-CASSCF and XMS-CASPT2 methods (see the Supporting Information).

We begin our investigation by focusing on the photoabsorption cross-section of CF_3_COCl (see Figure 3) and understanding the energy and character of the low-lying excited electronic states of this molecule. Comparison between theory and experiment reveals that the low-energy band (220–320 nm) can be attributed to a transition to the first excited electronic state (*S*_1_), which exhibits a n_CO_ → π_CO_* character (see the right panel of Figure 3). This character is consistent with the low-absorption cross-section observed for this first band. Moving to shorter excitation wavelengths leads to population of the *S*_2_ and *S*_3_ electronic states of CF_3_COCl. Both electronic states are close in energy (see Figure 2) as they are of p_Cl_ → π_CO_* character (right panel of Figure 3). At the optimized ground-state geometry, the *S*_2_ electronic state exhibits a p_*x*,Cl_ → π_CO_* character (with a sizable oscillator strength of 0.0166), while *S*_3_ shows a p_*y*,Cl_ → π_CO_* character resulting in a smaller oscillator strength (0.0006)—the two electronic states being separated by only 0.05 eV. In agreement with the experimental cross-section, theory shows that the second band in the photoabsorption cross-section (with a mixed contribution of transitions to the *S*_2_ and *S*_3_ states—molecular distortions altering the energy of the p_*x*,Cl_ → π_CO_* and p_*y*,Cl_ → π_CO_* characters) possesses a much higher intensity. The quantum yields of both products are also plotted in Figure 3 to explicitly highlight the wavelength-dependence. It shows the abrupt change of quantum yields in the middle of the first absorption band, which is of interest in this work.

The low-energy band of CF_3_COCl is thus only caused by the n_CO_ → π_CO_* transition, while the second absorption band—which is well separated from the first one—is due to transitions to the *S*_2_ and *S*_3_ states. Hence, using an excitation wavelength of 230, 235, 254, or 280 nm (black sticks in Figure 3) will promote CF_3_COCl exclusively in its *S*_1_ (n_CO_ → π_CO_*) excited electronic state, but with a different internal energy. Employing an excitation wavelength of 193 nm will excite the molecule into the *S*_2_ or *S*_3_ state and trigger a dynamics mediated by a p_Cl_ to π_CO_* transition. In the following, we propose to excite CF_3_COCl at these specific wavelengths and explore, both experimentally and theoretically, the resulting formation of photoproducts.

### 3.2 Photodissociation Following Excitation into the *S*_1_ State: 230–280 nm

Based on our earlier analysis of the photoabsorption cross-section, exciting CF_3_COCl between 230 and 280 nm is expected to populate the *S*_1_ (n_CO_ → π_CO_*) electronic state of this molecule. Here, we investigate the possible photoproducts that can be formed following photoexcitation.

**Figure 4:**
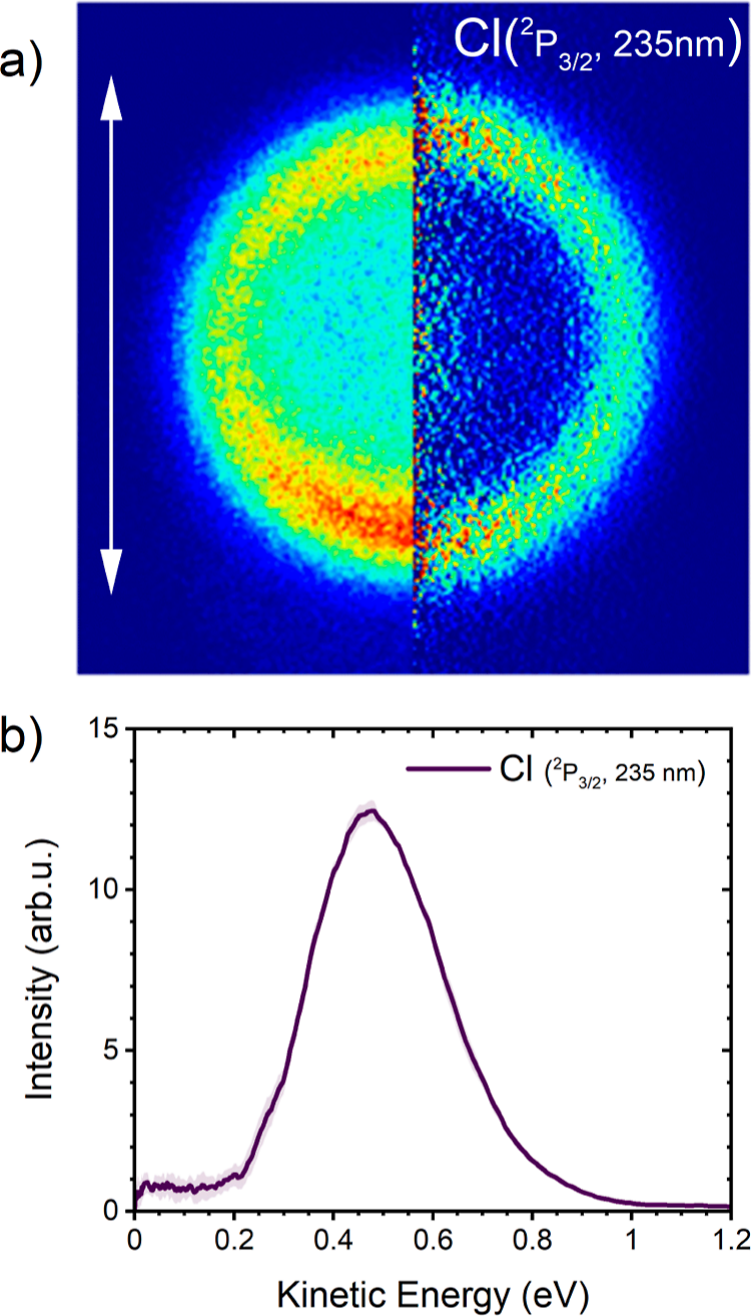
Acquired VMI image of Cl fragments after the single-color experiment at 235.31 nm (left, raw image; right, Abel transformed image) (a) and the corresponding KED spectrum (b). The angular distribution shown in Figure S10a of the Supporting Information yielded the fitted β-parameter β(Cl(^2^P_3/2_)) = 0.28(8). The white arrow in (a) indicates the orientation of the photolyzing laser polarization.

The Cl fragments can be REMPI ionized at 235 nm, as outlined in the experimental section. Thus, we first perform a single-color experiment at this wavelength. The acquired image of Cl(^2^P_3/2_) fragments is shown in panel (a) of Figure 4 with the corresponding KED spectrum in panel (b). The angular distribution is shown in Figure S10a. The single ring structure with a slight parallel anisotropy character indicates delayed ballistic ejection of the Cl(^2^P_3/2_) fragments after the excitation. The fragment kinetic energy is centered at 0.48 eV with a full width at half-maximum (fwhm) of 0.31 eV. The fastest detected ions can reach kinetic energies up to 1 eV. The slight anisotropy of the ring is revealed by the fitted anisotropy parameter β = 0.28 ± 0.08. Since a single electronic state is involved in the excitation, the departure of the β value from 2 for the parallel transition corresponds to the delayed ejection of the fragment due to a relatively long dwelling time after the UV excitation in the shallow minimum in the Franck–Condon region of the *S*_1_ state. Analogous measurement was done with the laser tuned at the Cl(^2^P_1/2_) REMPI wavelength. The detected signal is approximately six times lower compared to the ground Cl signal. Nevertheless, the obtained images exhibit the same KED distribution and anisotropy character (see Figure S11).

**Figure 5:**
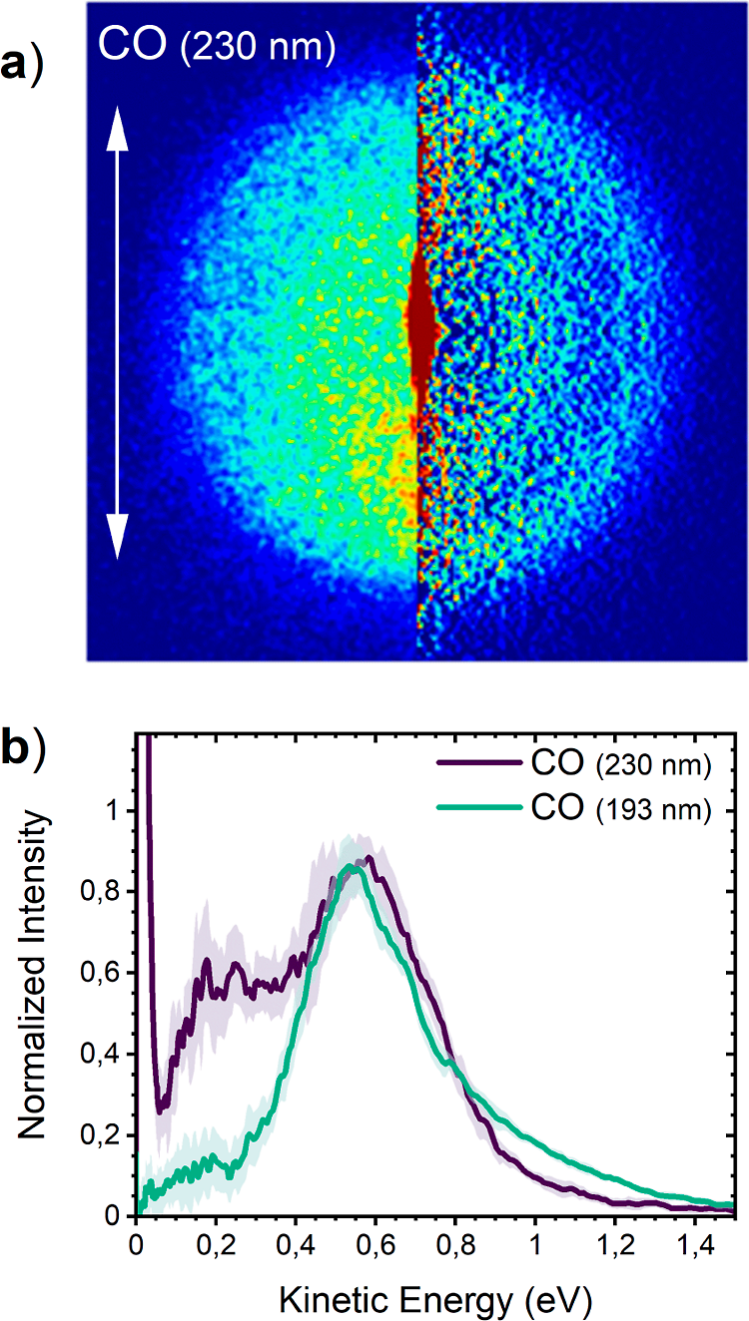
Acquired VMI image of CO(ν = 0) fragments after the single-color experiment at 230.046 nm (left: raw image; right: Abel transformed image) (a) and the corresponding KED spectrum (blue) and angular distribution (b). The KED of the CO fragments taken after photodissociation at 193 nm (green) is shown for comparison. [The corresponding angular distribution for the fragments with *E*_kin_ > 0.4 eV is shown in Figure S10b; β(CO, *E*_kin_ > 0.4 eV) = 0.36(10)].

The CO(ν = 0) fragments can be REMPI ionized at 230.046 nm. Thus, a single-color experiment at this wavelength shows the VMI image of the CO fragments in Figure 5. The signal is weaker, as indicated by the significantly larger error bars on the corresponding KED. An outer ring of the fast CO fragments is accompanied by a broad distribution of slower fragments and a sharp central feature. The sharp central feature with a much higher intensity corresponds to the signal from CO impurity in the primary beam. This is caused by a thermal decomposition of the CF_3_COCl molecules in the reservoir, and the longitudinal shape of its image corresponds to the primary beam velocity profile, as demonstrated previously, e.g., for HBr.^21^Figure 5b reveals the bimodal character of the KED of CO ion signal from the photofragmentation of CF_3_COCl at 230 nm. The faster fragments peak around 0.56 eV (fwhm ∼0.34 eV) and the maximum of the slower fragments occurs around ∼0.2 eV with a lower intensity. The angular distribution shown in the Supporting Information (Figure S10b) obtained by integrating only the CO fragments with kinetic energy above 0.4 eV yielded a β-parameter of 0.36(10) corresponding to the β-parameter obtained for the Cl(^2^P_3/2_) fragment.

Similar photofragmentation experiments were performed with excitation wavelengths of 254 and 280 nm. The corresponding KEDs are shown in Supporting Information Figure S13. The Cl(^2^P_3/2_) fragments show a single-ring structure independent of the photodissociation wavelength. We observe slight energy shift from 0.48 eV (fwhm ∼0.31 eV) at 235 nm to 0.44 eV (fwhm ∼0.22 eV) and 0.41 eV (fwhm ∼0.16 eV) at 254 and 280 nm, respectively.

### 3.3 Photodissociation Following Excitation into the *S*_2_/*S*_3_ States: 193 nm

Let us now focus on the photodynamics resulting from the excitation of CF_3_COCl at 193 nm, that is, in its second absorption band corresponding to the p_Cl_ → π_CO_* states (*S*_2_ and *S*_3_).

**Figure 6:**
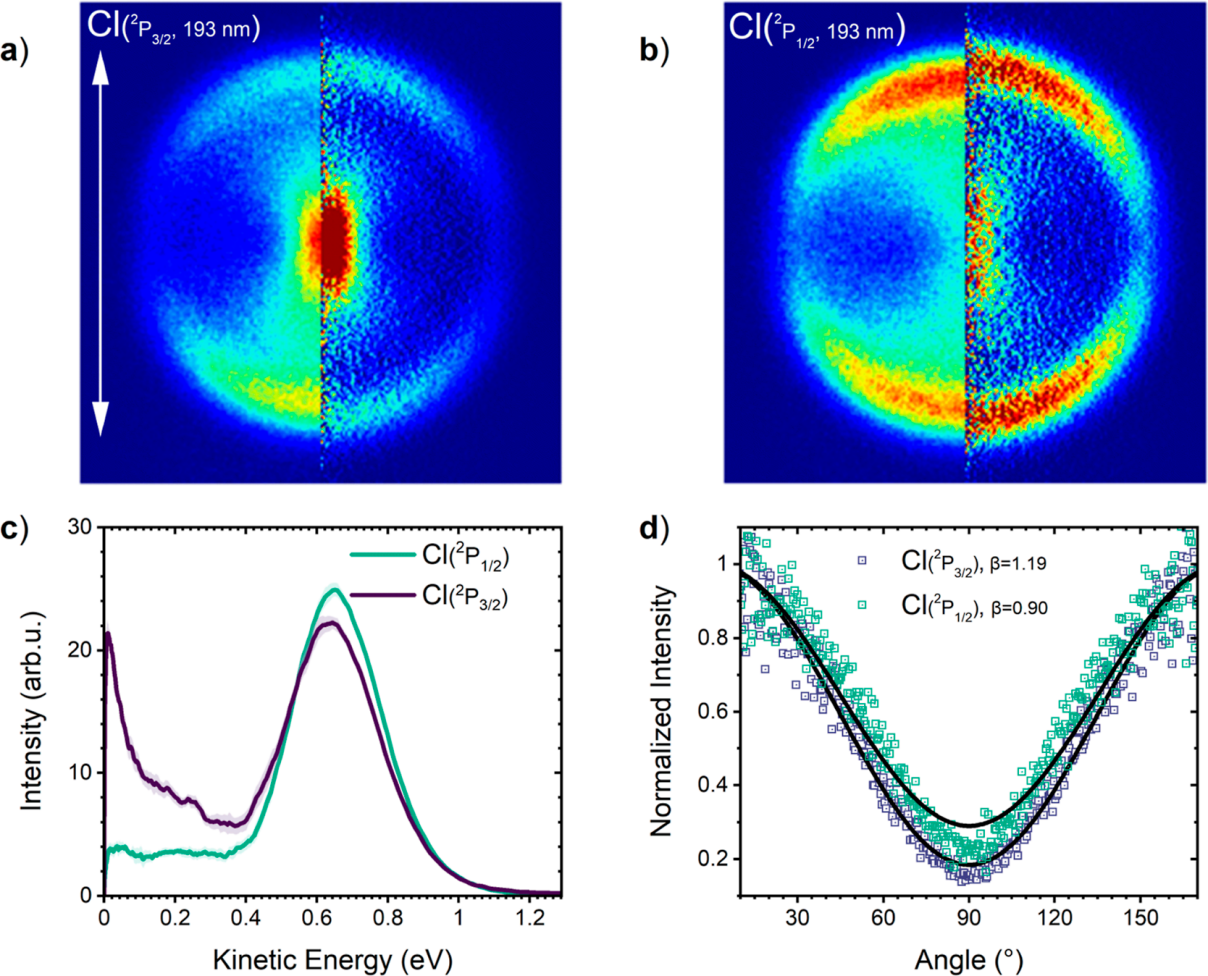
Acquired VMI images of Cl(^2^P_3/2_) (red) and Cl(^2^P_1/2_) (green) fragments after photodissociation at 193 nm and the corresponding KED spectra and angular distributions. The angular distribution shown in (d) shows only fragments with *E*_kin_ > 0.4 eV. The fitted β-parameters are β(Cl(^2^P_3/2_), *E*_kin_ > 0.4 eV) = 1.19 and β(Cl(^2^P_1/2_), *E*_kin_ > 0.4 eV) = 0.9 for Cl(^2^P_3/2_) and Cl(^2^P_1/2_) fragments, respectively.

The images of Cl(^2^P_3/2_) and Cl(^2^P_1/2_) fragments after the photodissociation at 193 nm are shown in Figure 6a,b, respectively. They exhibit a relatively sharp ring structure with a more pronounced anisotropy compared to the longer-wavelength photodissociation above. In addition, there are some central features. The contribution of the slow Cl(^2^P_1/2_) fragments is relatively low. On the other hand, the image of Cl(^2^P_3/2_) fragments exhibits a strong central intensity. In VMI, a feature near the center of an image is somewhat tricky to interpret, as the slow fragments can arise from the photodissociation of a molecule as well as from clusters or from multiphoton effects. Many examples exist in the literature^24,43^ (including some misinterpretations), and therefore, we carefully check for the multiphoton effects in the present investigation. The experiments performed at different excimer laser powers at 193 nm between 150 and 600 μJ/pulse are shown in Figure S12. Any clustering effects have been excluded as well, based on the expansion pressure and concentration dependence and the mass spectra. Thus, the central feature in Figure 6a corresponds to the Cl(^2^P_3/2_) fragment from the single-photon dissociation of the CF_3_COCl molecule, and the elongated shape results from the convolution of the low-energy fragment velocity with the beam velocity distribution, as suggested by the strongly elongated sharp peak in the center of the CO image in Figure 5.

The evaluated KED spectra are shown in Figure 6c. Independently of the detected Cl quantum state, they show a pronounced peak of faster fragments with energies >0.4 eV. The fitted maxima of the peaks have an energy of 0.61 eV (fwhm ∼0.28 eV) for the Cl(^2^P_3/2_) fragments and 0.64 eV (fwhm ∼0.28 eV) for the Cl(^2^P_1/2_) fragments. The KED of the Cl(^2^P_3/2_) fragments shows an additional increase of the signal toward zero corresponding to the central feature in the image. The Cl(^2^P_1/2_) fragments exhibit the slow fragments as well, but at a lower abundance with a flat distribution between 0 and 0.4 eV. The angular distribution of both Cl(^2^P_3/2_) and Cl(^2^P_1/2_) fragments (>0.4 eV) shows a strong anisotropy with fitted β-parameters of 1.19 and 0.9, respectively. The energy at 193 nm (6.41 eV) excites higher dissociative excited states from which the Cl fragment is immediately ejected.

We also recorded the VMI images of CO fragments at 193 nm. The obtained KED is shown in Figure 5b above, in comparison with the KED spectrum at 230 nm. At 193 nm, the KED is dominated by a peak at 0.55 eV. There is still a small contribution of the low-kinetic-energy fragments. However, its relative intensity is much weaker compared to that at 230 nm. Although there is a somewhat higher contribution of the high-kinetic-energy fragments above 0.8 eV at 193 nm, the maximum position of the fast peak is almost independent of the photon energy. This and a comparison with the Cl KEDs above suggest a sequential three-body fragmentation at 193 nm. It is further discussed with the outcomes of the *ab initio* simulations.

### 3.4 Mapping the Potential Energy Surfaces of CF_3_COCl

We inspected the potential energy curves along the Cl and CO dissociation coordinates with the XMS-CASPT2 method. This provides us with the first ideas about possible mechanisms consistent with the VMI experiments.

**Figure 7:**
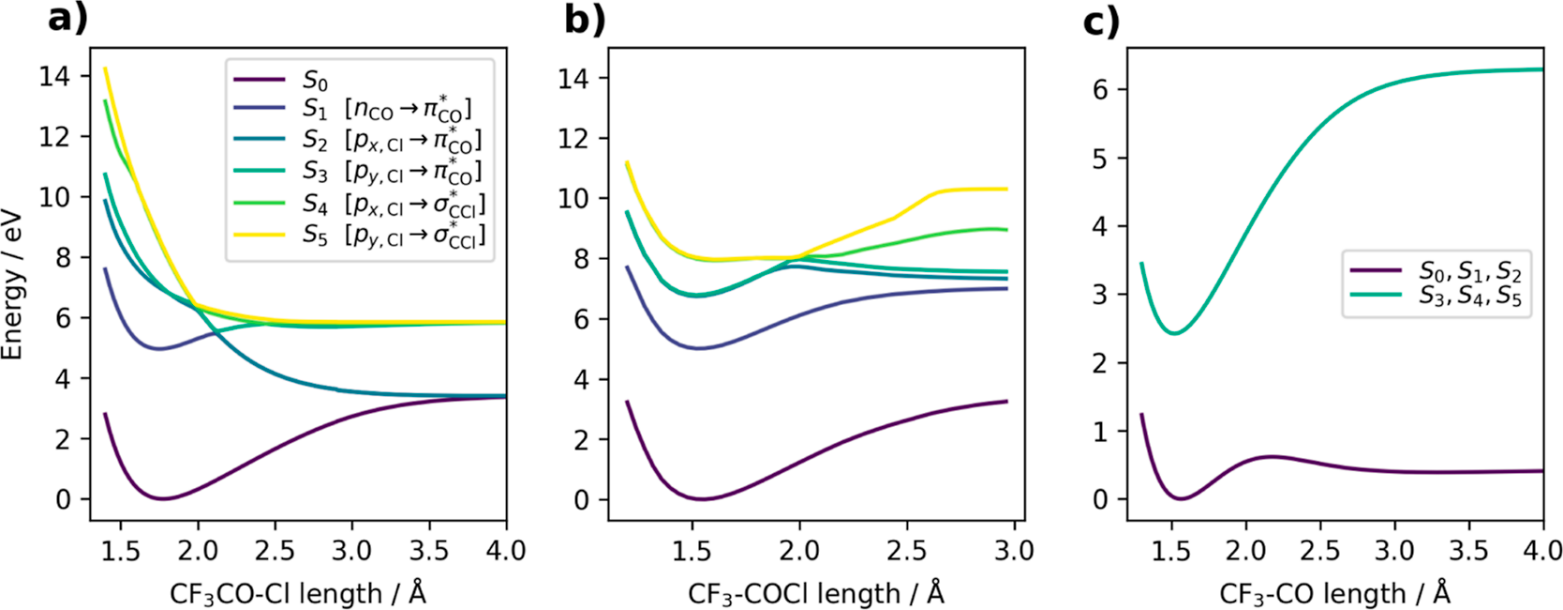
Rigid scan of electronic energies along the (a) CF_3_CO–Cl, (b) CF_3_–COCl, and (c) CF_3_–CO dissociation coordinates obtained at the XMS-CASPT2(12,9) level. The results for (b) are presented only up to 3 Å as the perturbation procedure ran into divergence problems for longer bond lengths. This issue can be alleviated by extended dynamical weighting, which confirms the presented picture; see the Supporting Information.

Figure 7a shows the electronic energies along the Cl dissociation coordinate. We see that upon excitation to the first excited state, the molecule is subject to a barrier appearing via the interaction with the higher excited state. When the barrier is overcome, the molecule is driven toward dissociation to the lowest dissociation limit. Contrarily, the excitation to *S*_2_/*S*_3_ is followed by immediate barrierless dissociation, with both dissociation limits energetically available. Populations of the higher and lower dissociation limits depend on the efficiency of nonadiabatic transitions. Note that *S*_2_ and *S*_3_ are always nearly degenerate and behave similarly since they correspond to excitation from degenerate p_*x*,Cl_ and p_*y*,Cl_ to π_CO_* (transition orbitals depicted in Figure 3). Figure 7b then shows the potential energy curves along the C–C bond in CF_3_COCl where the COCl fragment is released. The curves suggest that the molecule is always excited into a minimum for all low-lying states along this coordinate with high dissociation barriers. Note that a barrier was observed on the S_1_ state in many other aldehydes and ketones^44^ that might, however, stem from the use of single-reference methods for the dissociation reaction. On comparing them with the C–Cl bond, we can assume the Cl to be the first dissociating fragment since the repulsive state along the C–C bond is positioned energetically higher than the C–Cl repulsive state. The CO fragment then subsequently dissociates from the remaining CF_3_CO radical. Figure 7c captures the potential energy curves along the CO release coordinate from the CF_3_CO radical. While the dissociation from the upper state is highly endergonic, the dissociation in the ground state is subject to an approximately 0.69 eV barrier and is controlled by the excess of energy in the CF_3_CO radical after the nonadiabatic processes. The calculated activation barrier of 0.69 eV is in agreement with the value extracted from experimental data; see the Supporting Information.

To benchmark the electronic energies, we recalculated the dissociation energies using the CCSD(T)/6-31+G** method. The calculated dissociation energy for the Cl release was 3.36 eV, which is very close to the 3.44 eV of the XMS-CASPT2 method. The inclusion of the zero-point energy decreases the energy to 3.26 eV. For the CO dissociation from the CF_3_CO fragment, the energy was 0.23 eV for CCSD(T) and 0.42 eV for XMS-CASPT2. Including the zero-point energy into CCSD(T), we get an even lower value of about 0.13 eV. The discrepancy between XMS-CASPT2 and CCSD(T) is overall small and should not cause a large difference in the nonadiabatic dynamics. For more details, see the Supporting Information.

### 3.5 KEDs—a Comparison between Nonadiabatic Dynamics Simulations and Experiment

#### 3.5.1 *S*_2_/*S*_3_ Dynamics Following Photoexcitation at 193 nm

**Figure 8:**
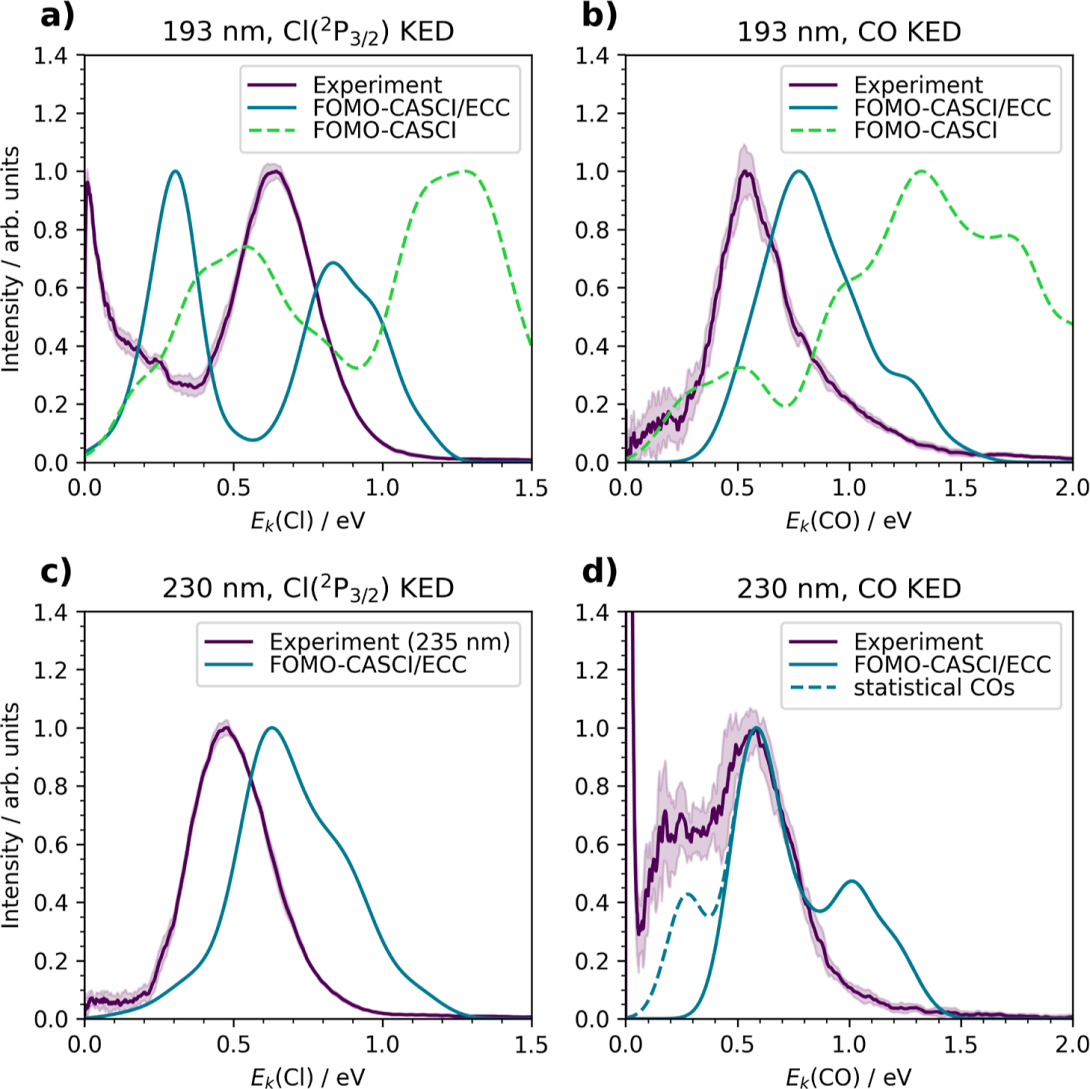
KEDs from experiment and simulations of (a) Cl(^2^P_3/2_) at 193 nm, (b) CO fragments at 193 nm, (c) Cl(^2^P_3/2_) fragments at 230 nm (compared to 235 nm experiment), and (d) CO fragments at 230 nm. The dashed line in (d) represents the estimated statistical release of CO.

Connecting the nonadiabatic dynamics simulations with the VMI experiments requires the calculation of experimental observables. In the following, we discuss the calculated KEDs (Figure 8). Let us first focus on the 193 nm excitation wavelength. The simulations were initiated from 200 points in the phase space: 100 in both the *S*_2_ and *S*_3_ states. All simulations led to Cl dissociation, followed by CO dissociation; some of the trajectories failed in this second step due to the electronic structure convergence problems. Note that Cl in calculations always refers to Cl(^2^P_3/2_) since we did not include the spin–orbit coupling. Both the Cl(^2^P_3/2_) and the CO fragments were released on average within 136 ± 74 fs. The KED of the Cl(^2^P_3/2_) fragment exhibits a two-peak structure, which is in line with the experimental measurements, although the peaks are shifted by about 0.25 eV to higher energies, see Figure 8a. This is partially caused by an excess of the excitation energy, since we applied a shift of 0.15 eV when selecting initial conditions in order to match the theoretical and experimental wavelengths. Another reason might be the inaccurate rigidity of bonds in the electronic structure method, causing a lower effective mass of the dissociating fragments. Such an effect was previously observed, e.g., for alkyl-halogenides.^25^ Nevertheless, increasing the rigidity of the structure did not affect the positions of the peaks (Figure S7). The ECC makes an important contribution, the kinetic energies at the pure FOMO-CASCI level deviate from the experiment significantly, yet the timescale remains the same (123 ± 71 fs).

Focusing on the mechanism, the two-peak structure originates from two dissociation limits available from the *S*_2_/*S*_3_ states. The low-energy peak represents a dissociation into the upper dissociation limit corresponding to an excited CF_3_CO fragment (n_CO_ → π_CO_*). From the excited state, the CF_3_CO fragment undergoes a fast internal conversion to the ground state via a conical intersection. The high-energy peak corresponds to the lower dissociation limit, releasing a Cl(^2^P_3/2_) atom and hot ground-state CF_3_COCl. In both cases, a hot ground-state CF_3_CO is formed, and the CO is then released. We note that both *S*_2_ and *S*_3_ states were degenerate during the dynamics and exhibited the same behavior throughout all dynamics. The CO KED exhibits a single peak structure and matches the experimental spectrum nicely but shifted again by 0.25 eV; see Figure 8b. The effect of ECC is again quite significant, and it also influences the shape of the peak in the present case. More details on the effect of ECC in simulations are presented in the Supporting Information.

#### 3.5.2 *S*_1_ Dynamics Following Photoexcitation at 230 nm

For the 230 nm wavelength, the dynamics were initiated from 100 initial conditions starting in the *S*_1_ state corresponding to the experimental excitation wavelength. Nevertheless, the KED spectra of Cl(^2^P_3/2_) and CO were constructed only from 39 trajectories because only these trajectories dissociated within the simulation time without suffering numerical instabilities. These instabilities appeared more frequently as the dynamics took much longer time than for the higher states due to the energy barrier on the *S*_1_ state. The calculated Cl(^2^P_3/2_) KED values are presented in Figure 8c. We compared it to the experimental KED measured at the one-color experiment at 235 nm since this is a relatively small energy difference. Again, the theory and experiment agree well within 0.25 eV.

For the CO KED, the comparison with the experiment is shown in Figure 8d. The dynamics confirmed the proposed mechanism of the Cl atom dissociating first, followed by the dissociation of CO from the ground-state CF_3_CO fragment. The CO dissociation usually followed only a few fs after the Cl release. A two-peak structure of the calculated KED is an artifact caused by the small number of analyzed trajectories since the mechanism leading to each peak is the same. The energy of the main peak is in good agreement with the experiment, yet the calculations do not reproduce the low energy peak in the experimental KED. This observation should not come as a surprise, since the slow fragments apparently originate from slow dynamics where the molecule evolves in the ground electronic state and dissociates at much longer times than achieved in our calculations. The experiment can capture CO fragments that dissociated up to 10 ns after the photon absorption.

To estimate the KED of the slow CO fragments, we assumed that the total kinetic energy available for dissociation is the difference between the energy of the transition state and dissociation limit [0.38 eV at the XMS-CASPT2(5,5) level; see the Supporting Information]. This leads to an approximate energy of 0.27 eV for the statistical release of CO. The intensity was taken as a sum of the absorption intensities of all trajectories staying in the CF_3_CO ground state, i.e., those that are subject to a statistical CO release. The resulting CO distribution is plotted as a dashed line in Figure 8d, which is quite in agreement with the experiment.

## 4 Wavelength Dependence of the CF_3_COCl Photochemistry

**Figure 9:**
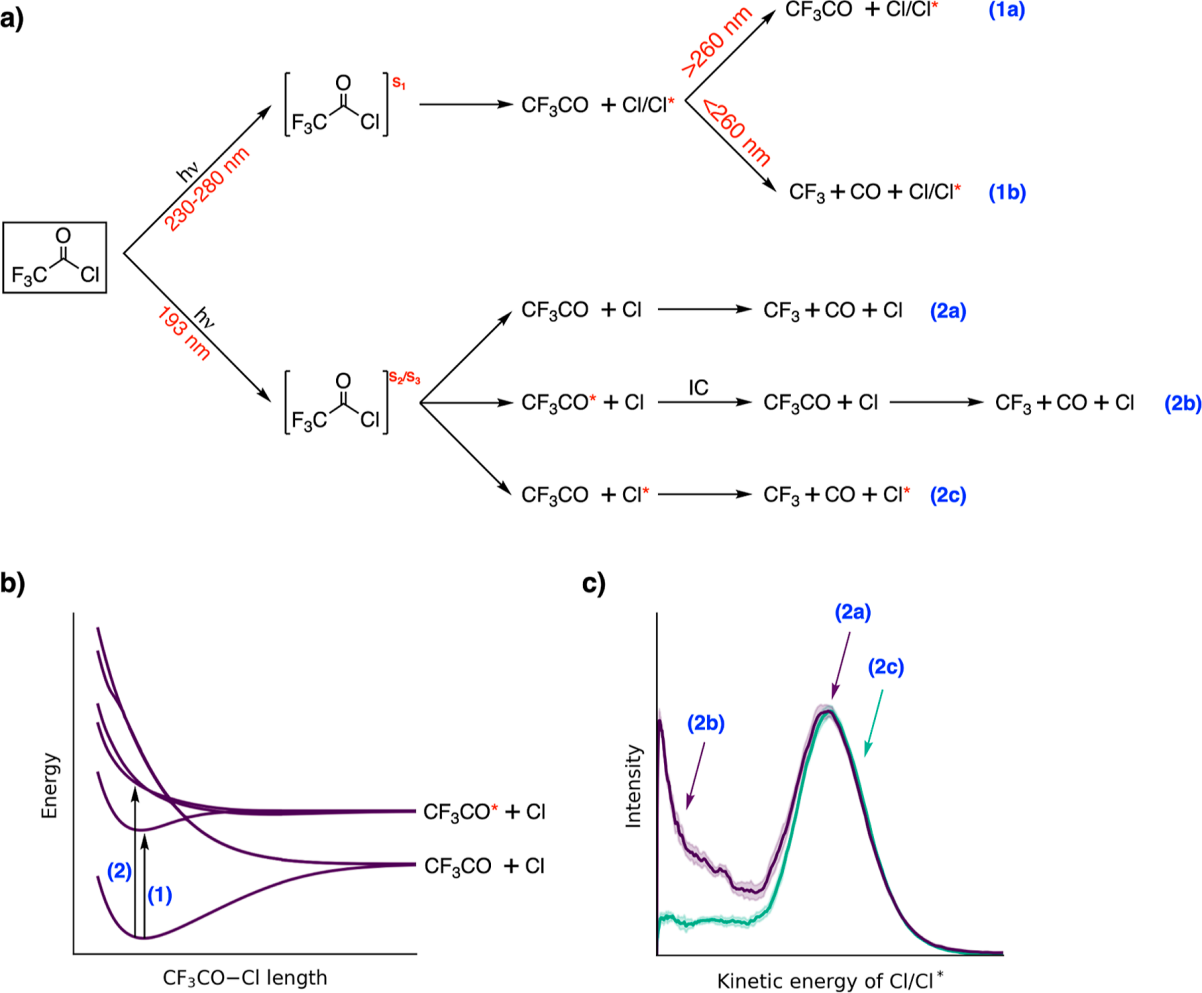
Reaction scheme of the CF_3_COCl photodecomposition mechanism as proposed in this work. We refer the ground spin–orbit state Cl(^2^P_3/2_) as Cl and the excited spin–orbit state Cl(^2^P_1/2_) as Cl*.

The most remarkable fact about the photodissociation of the title molecule is its wavelength-dependent photochemistry, even within a single UV band. Our suggested mechanism is presented in Figure 9. The mechanism is different for excitation to the first absorption band (pathways 1a and 1b) at 230–280 nm (Figure 9) or the second one (pathways 2a, 2b, and 2c) at 193 nm (Figure 9). Excitation to the first band ends in the *S*_1_ state and is followed by a direct Cl dissociation (direct CO dissociation is energetically forbidden) leaving the hot ground-state CF_3_CO fragment behind. The subsequent dissociation of CO from CF_3_CO depends on the internal energy left in the fragment and the activation barrier. While CO dissociates for shorter wavelengths than 260 nm (1b), it gradually stops dissociating for longer wavelengths (1a).^5^ Hence, we observe wavelength-dependent photodynamics within one absorption band. As this molecule has a small number of nuclear degrees of freedom and is in the gas phase, the photodissociation of CO is ultrafast when energetically allowed.

The above-proposed mechanism is corroborated by the experiment. For the excitation wavelengths 235, 254, and 280 nm, the Cl fragments (meaning both ^2^P_3/2_ and ^2^P_1/2_ spin–orbit states) are observed and their KEDs exhibit a single peak with a weak parallel character. The fragment kinetic energy (0.4–0.5 eV at KED maximum) does not correspond to a direct two-body dissociation, which could be approximated by a hard sphere model.^25^ Such a model would yield Cl fragment kinetic energies of 1.48, 1.19, and 0.86 eV, respectively, for the above wavelengths [assuming *D*_0_(C–Cl) = 3.26 eV]. Thus, a relatively large part of the excitation energy is dissipated into the internal excitation of the CF_3_CO fragment (1.36, 1.02, and 0.61 eV, respectively, corresponding to the position of the KED maximum). This energy can lead to further dissociation of the fragment leading to the CO fragment observed at 230 nm.

The weak anisotropy of both Cl(^2^P_3/2_) and Cl(^2^P_1/2_) suggests that the dissociation is not immediate on the timescale of the title molecule rotation, and the delay can lead to energy redistribution. The similarity of the faster CO fragment anisotropy at 230 nm with the anisotropy of the Cl at 235 nm may suggest that the dissociation of the faster CO fragments follows Cl almost immediately. This view is supported by our simulations. The CO fragment KED at 230 nm also shows a contribution of slow fragments. Such slow fragments can be explained as a delayed statistical decay of CF_3_CO in the ground state, as suggested by our simulations. The anisotropy parameter for this process cannot be obtained reliably due to a lower signal in this region and strong overlap with the primary beam CO and fast fragments. Nevertheless, lower β values (close to 0) are obtained, as expected for a slow process averaged over the molecular rotation. Finally, no reasonable CO fragment signal can be measured at 254 and 280 nm, as expected for wavelengths above 250 nm.

The excitation to the second absorption band is more complex; see Figure 9. It promotes the molecule to either *S*_2_ or *S*_3_ which is nearly degenerate in the Franck–Condon region. The molecule is then immediately driven toward the Cl dissociation. For the Cl(^2^P_3/2_), the CF_3_CO fragment can end up in either its ground state (2a) or its excited state (2b). From the excited state (2b), the molecule undergoes a fast internal conversion to the ground state via a conical intersection. The dissociation of CO from excited CF_3_CO is improbable due to a large energetic barrier. In any case, there is always enough energy for the subsequent dissociation of CO. Following photoexcitation, the molecule can also end up in an electronic state, leading to a spin–orbit excited Cl(^2^P_1/2_) (2c). In such a situation, Cl(^2^P_1/2_) dissociates and leaves CF_3_CO behind in its ground electronic state—a spin–orbit-controlled dissociation. It is interesting to note the large effect of the very small spin–orbit coupling in chlorine. While there are reaction channels available for the ground spin–orbit state Cl(^2^P_3/2_), there is only one of them present for the excited spin–orbit Cl(^2^P_1/2_). This effect is probably caused by excitation into the crossings between the electronic states.

The proposed mechanisms of the 193 nm photodissociation are supported by experimental observations. The Cl(^2^P_3/2_) and Cl(^2^P_1/2_) produced at 193 nm exhibit a fast fragment peak with a maximum at about 0.6 eV, corresponding to channels (2a) and (2c). The shift in the energy with respect to 235 nm KED does not correspond to the difference in the excitation photon energy. It confirms that a significant portion (2.33 eV) of the available energy (3.164 eV) is deposited into the CF_3_CO fragment internal excitation and can lead to its dissociation yielding the CO fragment. At 193 nm, the ejection of the fast Cl fragments is a much faster process in comparison to what was measured at longer wavelengths, resulting in parallel anisotropy with a much higher β ≈ +1. The slow Cl fragments with near-zero kinetic energy originate from pathway 2b, where the energy is mostly taken by the excited CF_3_CO* fragment. Interestingly, this channel is operative only for the ground-state Cl(^2^P_3/2_), i.e., not spin–orbit excited. When the Cl is spin–orbit excited, the channel releasing slow Cl(^2^P_1/2_) fragments is suppressed, and only fast fragments are observed. Obtaining the anisotropy parameter for the slow Cl fragments is hampered by their image distortion due to the convolution with the primary beam, as discussed in the Results section. Therefore, the experiment does not provide any additional clue for the timescale of the slow Cl fragment production with respect to the fast ones.

The CO fragments at 193 nm exhibit predominantly a fast KED peak centered at 0.55 eV with anisotropy β ≈ 0.36 and a small contribution of slow fragments (*E*_k_ = 0.3 eV). The KED peaks are essentially located at the same energy as observed at 230 nm, but the slow fragments peak almost disappears at a 193 nm photoexcitation. These observations support the above suggestion that the dissociation of CO follows very shortly after the Cl, so that the energy is already partly redistributed, but the anisotropy of the CO fragment is not completely washed out by the CF_3_CO rotation yet.

We finally note that CF_3_COCl belongs to the same family as CF_3_COH, a refrigerant of the third generation. It has been recently argued that photolysis of this molecule leads to the formation of CHF_3_, one of the most potent greenhouse gases. While the photochemistry of CF_3_COH and CF_3_COCl might be expected to be similar, the quantitative differences between these two atmospheric molecules lead to a photochemical instability of the C–Cl bond even at long wavelengths, meaning that the analogical CF_3_Cl product only appears as a very minor product that could not be detected in our work.^45^

## 5 Conclusions

In this work, we unravel the complex photochemistry of CF_3_COCl, a prototypical molecule found in our atmosphere upon photo-oxidative degradation of halo-carbon substitutes. The mechanism exhibits several remarkable features. First, a relatively small spin–orbit coupling in chlorine was demonstrated to control the reaction yields for different channels. Second, the formation and destruction of the CF_3_COCl molecule depend sensitively on the UV wavelength in the ranges relevant to the upper troposphere and lower stratosphere. By combining VMI experiments and nonadiabatic *ab initio* molecular dynamics simulations, we showed that CF_3_COCl obeys different regimes of non-Kasha behavior, i.e., wavelength control of photochemical reactivity. More specifically, the photochemistry of CF_3_COCl exhibits a dual case with a wavelength dependence controlled simultaneously by (i) the internal energy deposited into the molecule within a single electronic state and (ii) the population of different electronic states. The energy range for these two regimes is relatively narrow, which should serve as a warning that wavelength-dependent photodynamics might be more important than expected in gas-phase photochemistry and may lead to the formation of a wider variety of (possibly unexpected) photoproducts. These findings may cast some shadow on the assumption that photoproduct quantum yields are wavelength-independent in the determination of photolysis rate constants for transient volatile organic compounds.

## Supporting Information Available

Discussion on the active space used, comparison between potential energy curves at several levels, discussion of the effective correlation correction, 2D potential energy scans, calculated dissociation energies, calculated photoabsorption cross-sections, experimental angular and KEDs of photodissociation fragments at different wavelengths, and photoionization time-of-flight mass spectra (PDF)
