# Supplementary material for: On the Wavelength-Dependent Photochemistry of the Atmospheric Molecule CF3COCl
Source: ACS Earth Space Chem. 2023 Oct 16;7(11):2275–86. doi: 10.1021/acsearthspacechem.3c00196 (PMC10658617; doi:10.1021/acsearthspacechem.3c00196)
Supplement: Supplementary file 1 — sp3c00196_si_001.pdf [file sp3c00196_si_001.pdf]

Supporting Information: On the wavelength-dependent photochemistry of the atmospheric molecule CF_3COCl

Jiří Janoš,[†] Ivo S. Vinklárek,[‡,§] Jozef Rakovský,[‡] Deb Pratim Mukhopadhyay,[‡,||] Basile F. E. Curchod,[¶] Michal Fárník,[*,‡] and Petr Slavíček[*,†]

†*Department of Physical Chemistry, University of Chemistry and Technology, Technická 5, Prague 6, 166 28, Czech Republic*

‡*Department of Dynamics of Molecules and Clusters, J. Heyrovský Institute of Physical Chemistry, v.v.i., The Czech Academy of Sciences, Dolejškova 2155/3, 182 23 Prague, Czech Republic*

¶*Centre for Computational Chemistry, School of Chemistry, University of Bristol, Bristol BS8 1TS, United Kingdom*

§*Center for Free-Electron Laser Science, Deutsches Elektronen-Synchrotron DESY, Notkestrasse 85, Hamburg, 22607, Germany*

||*Synchrotron SOLEIL, L'Orme des Merisiers, St. Aubin BP 48, 91192 Gif sur Yvette, France*

E-mail: michal.farnik@jh-inst.cas.cz; Petr.Slavicek@vscht.cz

Phone: +420 (2)6605 3206. Fax: +420 (2)6605 3910

1 Supplementary computational details

1.1 Limitations of the SA2-CASSCF(8,7) methodology to describe the photochemistry of CF_3COCl

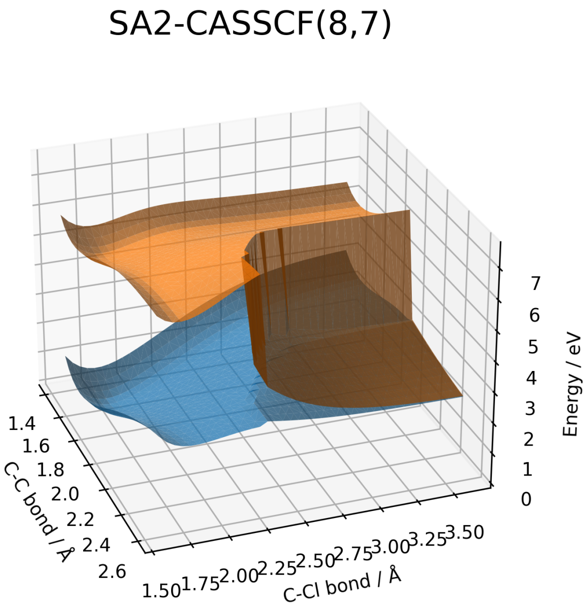

Figure S1: Electronic energies of the S_0 and S_1 states generated with SA2-CASSCF(8,7) (left) and SA6-CASSCF(12,9) method (right). The 2D scan goes along both dissociating coordinates: the C–Cl and C–C bonds.

Previously reported excited-state dynamics simulations of CF_3COCl were based on an SA2-CASSCF(8,7) level of electronic-structure theory.[S1] This active space lacks the $p_{\mathrm{Cl,y}}$ and $p_{\mathrm{Cl,x}}$ orbitals, which are important to capture the character of dissociated states because of the degenerate nature of the electronic states in the Cl dissociation. The SA procedure also requires four more excited states in order to capture the correct dissociation limit, since it is the S_4 and S_5 electronic states with p_{Cl} orbitals that become degenerate with the S_0 state upon Cl dissociation – S_1 becomes degenerate with S_2 and S_3. Without the inclusion of these orbitals and electronic states, the SA-CASSCF method results in artificial conical intersections and large total-energy discontinuities during the non-adiabatic dynamics. To demonstrate the issue of this active space and state averaging, we calculated a 2D scan of

the S_0 and S_1 states along the C-C and C-Cl bonds using the SA2-CASSCF(8,7) and SA6-CASSCF(12,9) methods (Figure S1). Excited-state dynamics with the SA2-CASSCF(8,7) methods led to failure in total energy conservation.

1.2 Comparing potential energy curves obtained with SA-CASSCF, XMS-CASPT2, and FOMO-CASCI

Three methods were applied for the electronic structure calculations: SA6-CASSCF(12,9), XMS-CASPT2(12,9), and FOMO-CASCI(14,11). Here, we present a benchmark of all these methods along the C–Cl, C–C, and CF_3–CO (Cl is kept dissociated) bonds (Figure S2). The results show that FOMO-CASCI offers a similar result to CASSCF for the relevant electronic states and can be used as a substituent. The dynamical correlation embedded in the XMS-CASPT2 retains the qualitative picture of the potential energy curves but changes the dissociation energy and activation barrier in the S_1 state. These changes are essential for capturing the correct kinetic energy of outgoing fragments and the time scale of the processes in the S_1 state.

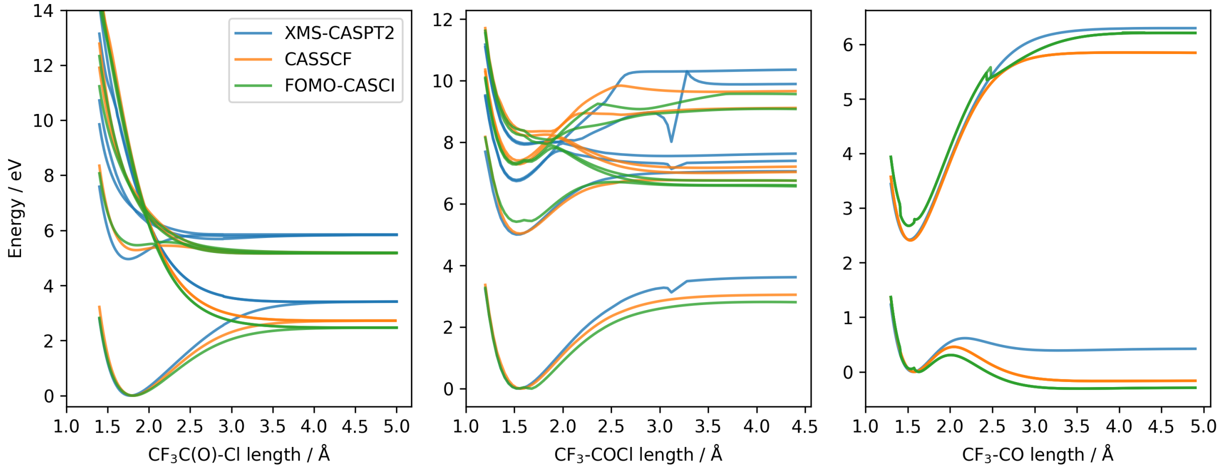

Figure S2: Benchmark of electronic energies obtained with XMS-CASPT2(12,9), SA6-CASSCF(12,9), and FOMO-CASCI(14,11) for different bond elongations.

1.2.1 CF_3–COCl scan with XMS- and XDW-CASPT2

As discussed in the main text, the XMS-CASPT2 electronic energies along the CF_3–COCl coordinate diverge beyond 3 Å leading to the appearance of a discontinuity, even if the reference SA-CASSCF curves are smooth (see Figure S2 above). This behavior can be improved by employing an extended dynamically weighted (XDW) formalism, which is a hybrid approach combining the best of XMS- and MS-CASPT2.[S2] The electronic energies calculated with the XDW-CASPT2 method show no discontinuities, even though the potential energy curves are not perfectly smooth (Figure S3). In the dissociation limit, XDW-CASPT2 predicts slightly different electronic energies, even if the corresponding electronic states retain the same character as in XMS-CASPT2 and do not contradict the deduced mechanisms.

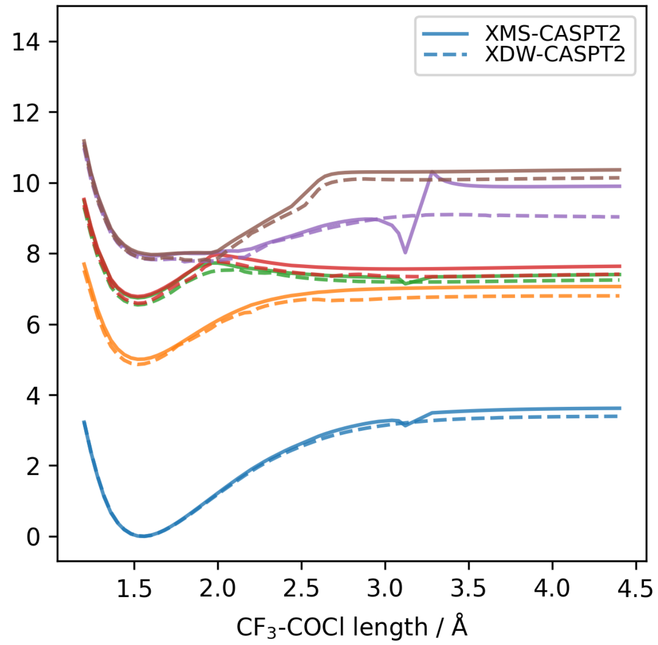

Figure S3: Electronic energies along the CF_3–COCl coordinate obtained with XMS-CASPT2 (solid lines) and XDW-CASPT2 (dashed lines).

1.3 Effective correlation correction

Here, we provide details about the ECC employed in this work. The ECC is used to correct the FOMO-CASCI electronic energies such that they approach those of XMS-CASPT2. The main reason for applying this correction is to improve the dissociation energies, meaning more accurate KED can be obtained from the excited-state dynamics simulations.

A Morse potential was used for the ECC formula

$$ECC(r) = (D_e^{corr} + c)\left[1 - \mathrm{e}^{-a(r-b)}\right]^2 - c \tag{1}$$

with the corresponding force correlation correction

$$FCC(r) = -2a(D_e^{corr} + c)\left[1 - \mathrm{e}^{-a(r-b)}\right]\mathrm{e}^{-a(r-b)}\,, \tag{2}$$

where r is a bond length along which the ECC is applied, D_e^{corr} is the difference between XMS-CASPT2 and FOMO-CASCI dissociation energies (this parameter was not fitted) and a, b and c are the fitted parameters. The Morse potential was chosen because it has physically reasonable boundary conditions, and it reasonably fits the electronic-energy difference between FOMO-CASCI and XMS-CASPT2, as depicted in Figure S4. The procedure for creating the ECC is as follows. First, a rigid scan along a given coordinate is performed with FOMO-CASCI and XMS-CASPT2. The difference between the dissociation energies of XMS-CASPT2 and FOMO-CASCI (D_e^{corr}) is obtained. With this parameter fixed, the a, b, and c parameters can be fitted for a target electronic state. Finally, the mean square deviation $\langle \Delta E^2 \rangle$ and the mean deviation $\langle \Delta E \rangle$ are calculated to evaluate the quality of the fit.

The correction was applied along two dissociation coordinates: the C–Cl and C–C bonds. First, let us discuss the ECC obtained for the C–Cl dissociation coordinate. Two different strategies were attempted, (a) and (b), with corresponding results presented in Figure S4a

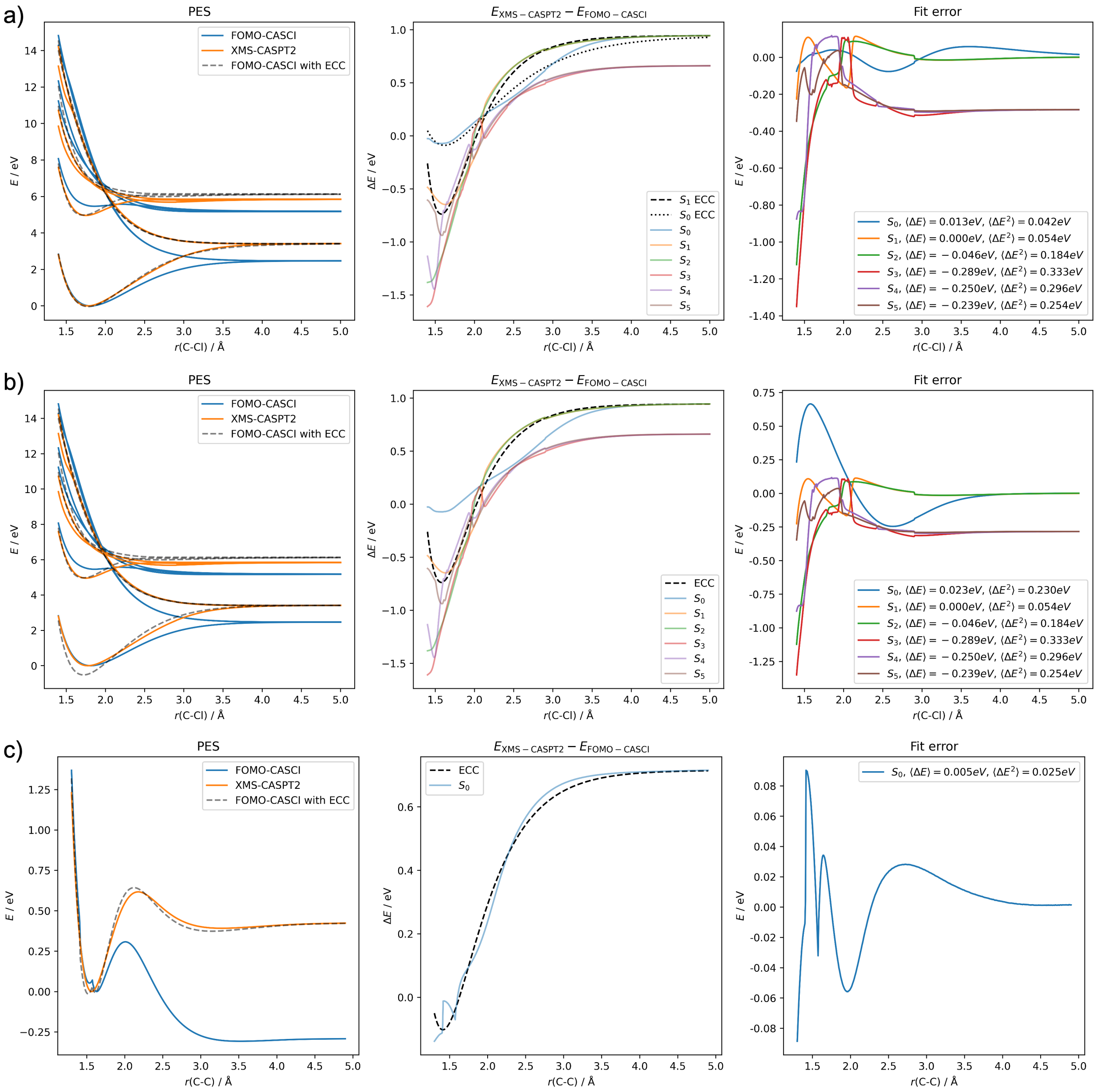

Figure S4: The ECC applied to FOMO-CASCI(14,11) to reproduce the XMS-CASPT2(12,9) results. (a) ECC for the C–Cl bond with different corrections for the ground and the excited states. (b) ECC for the C–Cl bond with the same correction for all the states. (c) ECC for the C–C bond in the CF_3CO fragment.

and S4b. (a) We employed separate corrections for the ground and the excited states, aiming at improving the absorption spectrum (Figure S4a). The D_e^{corr} was evaluated for the lower dissociating limit (between S_0, S_1, and S_2) and was the same for both fits in order to ensure that they match in the limit of dissociation. The correction for the excited states was fitted

on the S_1 state since it is the state of interest for all the excitation wavelengths targeting the first absorption band. Although the correction was fitted for S_1, it significantly improved the behavior of all the other excited states. The ground state needs to be fitted separately as it requires different parameters for a proper match with the other electronic states. The different correction for the ground state is mainly responsible for the improvements in the absorption spectrum. (*b*) The other strategy consisted in using the fit of the S_1 state for all electronic states, i.e., no special fit was used for the ground state (Figure S4b). In this case, only the ground-state ECC changes in comparison to the case (*a*). The $\langle \Delta E^2 \rangle$ value increases from 0.042 in case (*a*) up to 0.230 in case (*b*).

The fit corresponding to case (*b*) was used in the non-adiabatic dynamics, as the fit obtained with the strategy (*a*) led to the appearance of artificial conical intersections between S_0 and S_1. These artificial conical intersections appear when the C=O bond is elongated and the S_0 and S_1 states are around 0.5 eV apart at the FOMO-CASCI level, but they would cross when employing the ECC. Applying the same ECC for all electronic states ensures that there are no such artificial conical intersections. The change of ECC for the ground state should not make any difference since the Cl dissociation happens in the excited states, and the ground state is not involved.

The ECC was also applied to the C–C dissociation (Figure S4c). The fit was performed with the Cl dissociated – Cl was kept 10 Å away from the CF_3CO fragment – since it dissociates first and CO is released from the ground state of the CF_3CO photoproduct. Therefore, we solely fitted the ground state of the CF_3CO fragment. The ECC dramatically improves the FOMO-CASCI electronic energy, reaching the quality of XMS-CASPT2. In particular, the ECC makes that the CO dissociation is now correctly endothermic instead of exothermic within FOMO-CASCI. This correction is not expected to influence the CF_3COCl dynamics as it just strengthens the C–C bond, which would not dissociate before Cl is released anyway.

All the final parameters used in the ECC models and obtained from the fits discussed in

this section are provided in the main text.

1.4 2D scans along dissocative coordinates

2D rigid scans of the PESs along the CF_3CO–Cl and CF_3–COCl coordinates are presented in Figure S5. The potential energy curves presented in the main text are cut through such 2D scans. These 2D scans corroborate the conclusion on the Cl mechanism proposed in the main text: the S_1 and S_2 states favor the Cl release over the whole range of CF_3-COCl distances. Thus, the Cl tends to be released first while the CO remains attached to the CF_3 fragment.

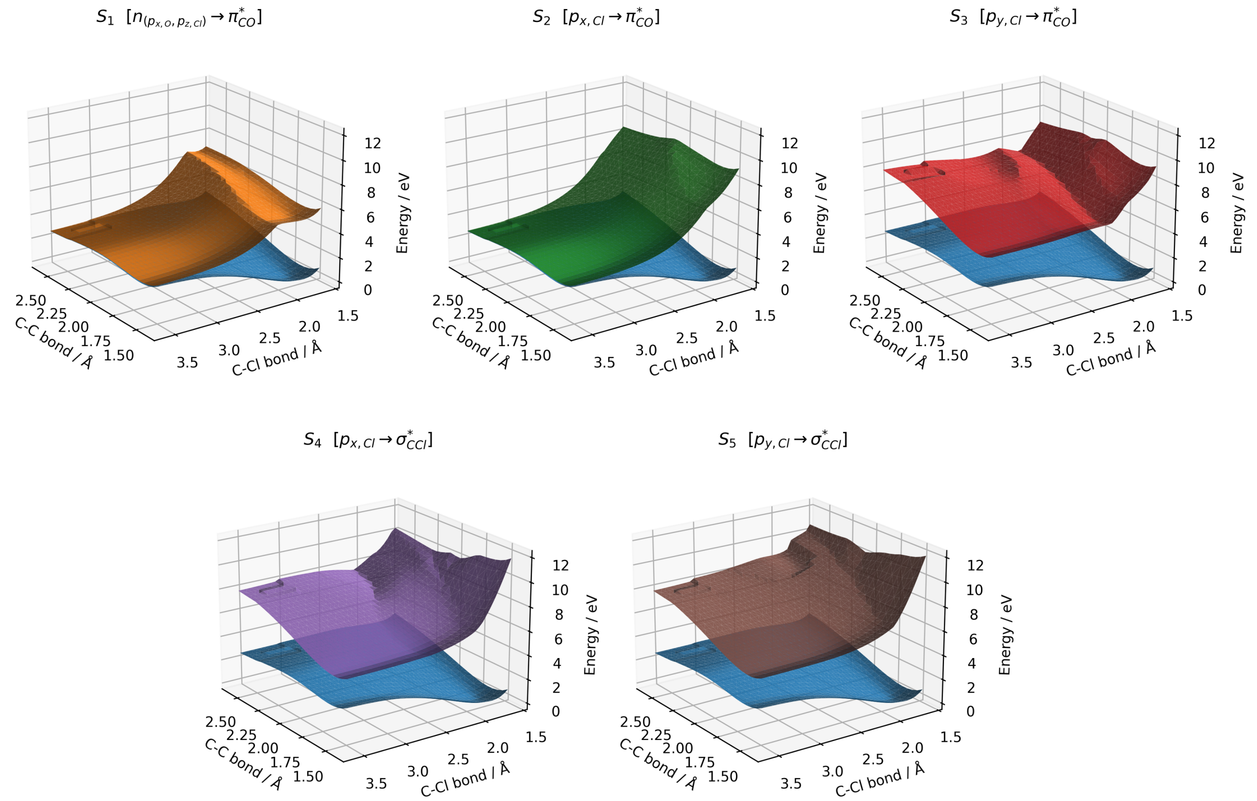

Figure S5: 2D scans along the CF_3CO-Cl and CF_3-COCl coordinates for the S_0-S_5 electronic states, calculated at the SA6-CASSCF(12,9) level. The ground-state (S_0) scan is provided in all panels, but each excited electronic state is plotted separately to improve clarity.

1.5 Dissociation energies

The 2D scans presented above are rigid, that is, obtained without a geometry reoptimization at each new point of the scan. As such, these plots can only provide an estimate of the dissociation energies. To determine accurate dissociation energies, we used optimized geometries and a high level of theory, CCSD and CCSD(T). The dissociation energies (D_e) and dissociation energies corrected with zero-point energy (D_0) for the Cl dissociation are presented in Table S1. The D_e predicted by CCSD(T) are in line with the XMS-CASPT2 values, while FOMO-CASCI underestimates the value of D_e by 1 eV. We note that the dissociation energies obtained with XMS-CASPT2 and FOMO-CASCI from the rigid scans are very close to those deduced from optimized geometries. Thus, the ECC fitting, based on rigid scans, should not introduce a large error. The zero-point energy correction calculated for CCSD and CCSD(T) decreases the energy by 0.1 eV.

Table S1: Dissociation energies for the $CF_3COCl \rightarrow CF_3CO + Cl$ reaction.

method	basis set	optimized	D_e^{gs} / eV	D_0^{gs} / eV	D_e^{ex} / eV
CCSD	6-31G*	✓	3.22	3.12	-
CCSD(T)	6-31+G**	✓	3.36	3.26	-
XMS-CASPT2(12,9)	6-31G*	✓	3.44	-	5.85
XMS-CASPT2(12,9)	6-31G*	✗	3.41	-	5.84
FOMO-CASCI(14,11)	6-31G*	✓	2.40	-	5.21
FOMO-CASCI(14,11)	6-31G*	✗	2.47	-	5.18

The dissociation energies D_e and D_0 for the CO dissociation from the CF_3CO fragment are presented in Table S2. The D_e predicted by CCSD(T) is lower by 0.2 eV than that obtained with XMS-CASPT2. However, both values show that the process is slightly endergonic. Conversely, FOMO-CASCI predicts that this dissociation is exergonic, with a D_e value of -0.51 eV. The unoptimized value obtained with XMS-CASPT2 and FOMO-CASCI from the rigid scans are again quite close to those calculated from optimized geometries – confirming again that the ECC fitting procedure based on rigid scans should not introduce a large error. The zero-point energy correction calculated for CCSD and CCSD(T) again decreases the energy by 0.1 eV.

Table S2: Dissociation energies of $CF_3CO \rightarrow CF_3 + CO$.

method	basis set	optimized	D_e^{gs} / eV	D_0^{gs} / eV
CCSD	6-31G*	✓	0.21	0.10
CCSD(T)	6-31+G**	✓	0.23	0.13
XMS-CASPT2(12,9)	6-31G*	✓	0.42	-
XMS-CASPT2(12,9)	6-31G*	✗	0.42	-
FOMO-CASCI(14,11)	6-31G*	✓	−0.51	-
FOMO-CASCI(14,11)	6-31G*	✗	−0.29	-

1.6 Photoabsorption cross-section

The photoabsorption cross-sections calculated with SA6-CASSCF(12,9), XMS-CASPT2(12,9), FOMO-CASCI(14,11), and FOMO-CASCI(14,11) with ECC levels are depicted in Figure S6. The cross-section obtained from FOMO-CASCI without ECC shows its first band blue-shifted by 0.59 eV, while the correction manages to correct the spectrum by 0.45 eV, in much better agreement with the experimental cross-section. The CASSCF and XMS-CASPT2 cross-sections serve as a reference to validate the use of FOMO-CASCI with ECC.

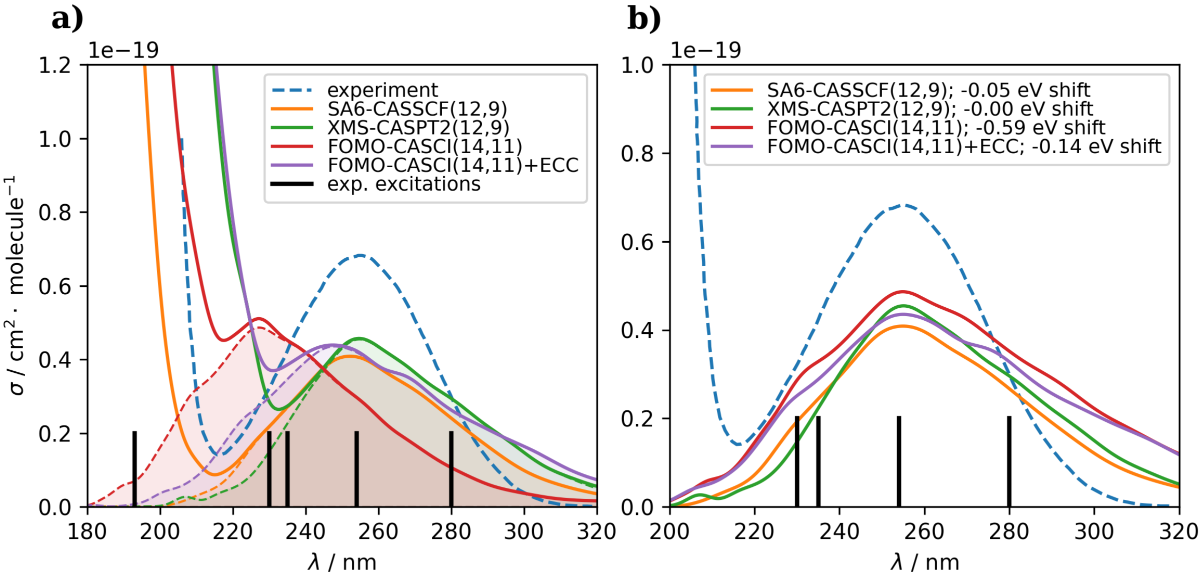

Figure S6: a) Calculated (solid lines) and experimental photoabsorption cross-section (dashed line, from Ref. S3). The colored area depicts the contribution from the S_1 state. b) The S_1 contribution to the calculated photoabsorption cross-sections, shifted to reproduce the experimental spectrum. Applied excitation wavelengths are depicted with black vertical lines in both panels.

1.7 KED with different ECCs for the excitation at 193 nm

To test the influence of the ECCs on the outcome of the simulations, we ran a series of FOMO-CASCI(14,11) excited-state dynamics with (*i*) no ECC, (*ii*) ECC applied only along the C–Cl bond, and (*iii*) ECC applied along both dissociation coordinates (Figure S7). The same set of initial conditions are used for all these dynamics and are the same as those employed for the calculations presented in the main text. (*i*) The results obtained with FOMO-CASCI significantly deviate from the experiment, with the peaks shifted by 0.4 to 0.8 eV (dotted lines in Figure S7). The shapes of the KED peaks also appear to be incorrectly reproduced by this level of theory, mainly for the CO KED (right panel of Figure S7). (*ii*) An ECC correction on Cl only improves the description of the Cl KED, shifting both peaks to lower energies, but with no overlap with the experiment (dashed lines in Figure S7). The intensity of these two peaks is not equal, as in experiment, but the higher energy peak exhibits a lower intensity. This feature might be probably attributed to the simple treatment of non-adiabatic transitions within the LZSH method, which governs the separation of the nuclear wavepacket in the dissociation limits. (*iii*) Surprisingly, the ECC applied to both Cl and CO leads to a degradation of the result for the first peak in the Cl distribution while leaving the other one unchanged. This shift of the first peak implies that the C–C mode is involved in the dissociation to the higher dissociation limit. As the ECC makes the C–C bond more rigid, more energy is put in the departing Cl atom. Conversely, the CO distribution is considerably improved with the CO ECC and reproduces the position and shape of the experiment.

Overall, our best simulations exhibit KEDs with a constant shift of about 0.25 eV from experimental values. An hypothesis to explain this deviation could be an incorrect rigidity of the bonds affecting the effective mass of the dissociated species. In other words, if the C–F bonds in the CF_3CO fragment are too soft, the effective mass of dissociating CF_3 will be almost equal to the mass of the C atom as the fluorine atoms are bound too loosely. If the bonds were more rigid, the effective mass would be higher and the partitioning of

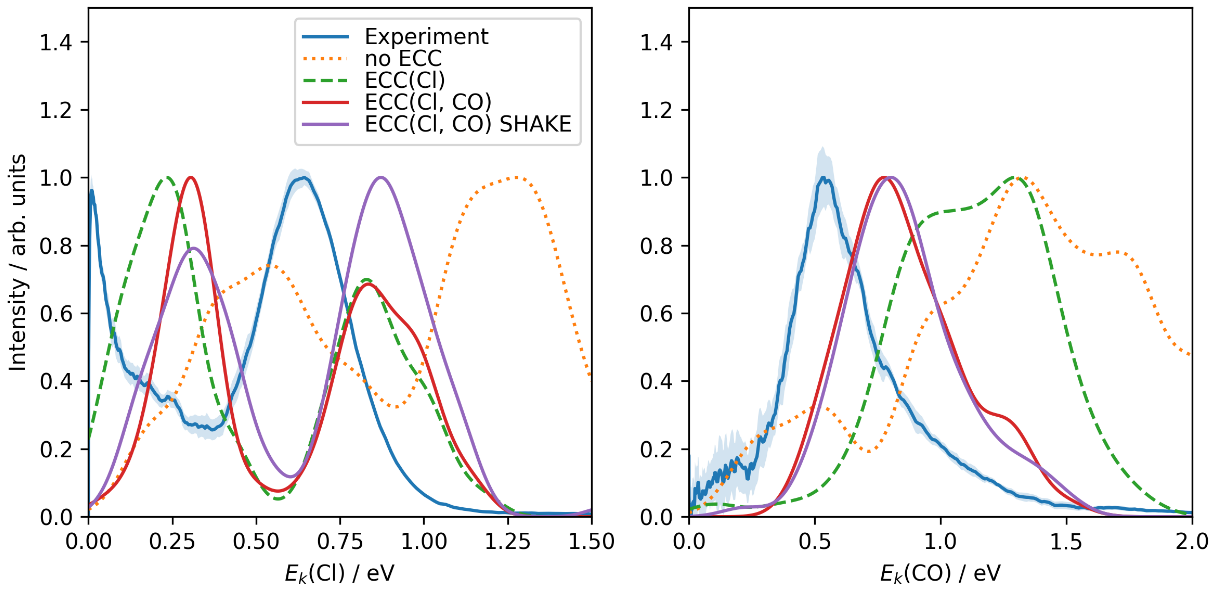

Figure S7: Kinetic energy distributions of Cl (left panel) and CO (right panel) fragments, calculated with different ECCs. ECC(Cl) shows results of excited-state dynamics with the ECC applied only along the C–Cl bond, while ECC(Cl, CO) shows the results obtained from simulation where the ECC is applied along both dissociating bonds, as described in Section 1.3. Simulations with the SHAKE algorithm considered all the C–F bonds as frozen. The experimental results is shown in both plots for comparison.

the energy would differ. To test that effect, we performed simulations with the FOMO-CASCI/ECC(Cl,CO) using the SHAKE algorithm to constrain the C–F bond lengths (Figure S7). Nevertheless, the position of the peaks remained unchanged, showing no effect of the bond constraint (the only difference lies in the change of relative intensities of the Cl KED, which points to an effect related to non-adiabatic transitions). The test simulations using SHAKE appear to refute the possible hypothesis for the shift of the KEDs, which originates from another source.

1.8 Activation barrier for CF_3CO dissociation

The CO dissociation barrier from the ground-state of the CF_3CO fragment was calculated from relaxed and rigid scans using the CASSCF and CASPT2 methods with active spaces ranging from (3,4) to (7,7) (Figure S8). The rigid scans along the F_3C–CO bond with the CASSCF method provides a range of activation barriers from 0.40 to 0.81 eV, depending on the active space (with no trends related to the size of the active space). The CASPT2 calculations yield a narrower range of 0.51-0.70 eV. Two active spaces – (3,4) and (7,6) – tend to lower values while (5,5), (5,6), or (7,7) active spaces predict a closer range of values, between 0.67 and 0.70 eV. The relaxed scan with the CASPT2(5,5) exhibits an activation barrier of 0.69 eV, which we consider as the best estimate value. The other active spaces suffered from convergence issues during optimization. To connect these results with our non-adiabatic dynamics, we also plot the rigid scan for the full CF_3COCl molecule, with the Cl atom kept 10 Å away. The activation barrier with XMS-CASPT2(12,9), which was used for the fitting of the ECC, is 0.46 eV. Therefore, the activation barrier in our non-adiabatic dynamics is about 0.2 eV lower than the predicted 0.69 eV.

1.8.1 Experimental value

The activation barrier can be extracted from the experiments combining the VMI data and quantum yields of CF_3CO.[S4] To do so, we calculated the internal energy distribution in the CF_3CO from the measured kinetic energy distribution of Cl ($E_{\mathrm{k,Cl}}$) with the energy conservation formula

$$E_{\mathrm{int}} = \mathrm{h}\nu - D_0 - E_{\mathrm{k,Cl}} - E_{\mathrm{k,CF_3CO}} = \mathrm{h}\nu - D_0 - (1 + \frac{m_{\mathrm{Cl}}}{m_{\mathrm{CF_3CO}}})E_{\mathrm{k,Cl}}\,, \tag{3}$$

where D_0 is the calculated dissociation energy of 3.26 eV, see Figure S9. The quantum yield of CF_3CO for each wavelength should correspond to the integrated internal energy distribution below the activation barrier (shaded areas in Figure S9). In other words, if

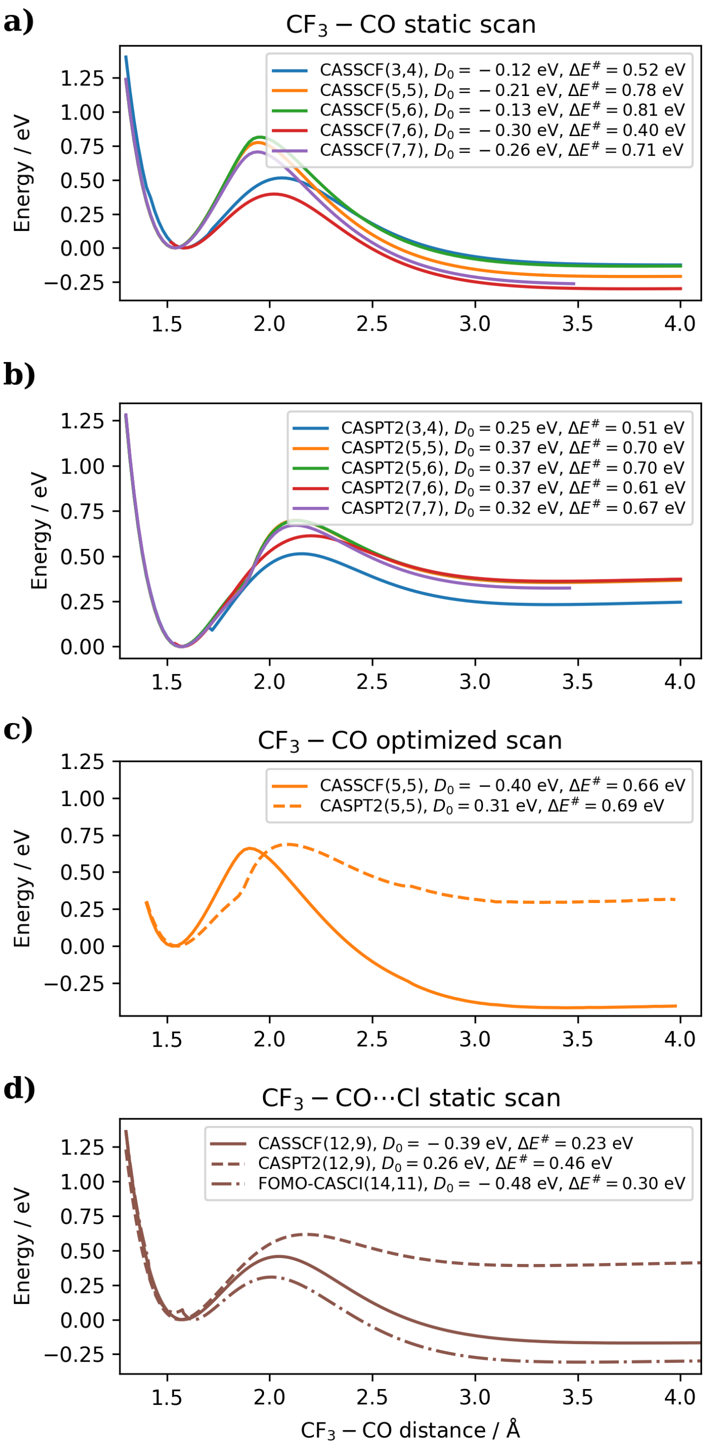

Figure S8: Scans along the C–C bond capturing the dissociation barrier of CO from CF_3CO. The dissociation energies D_0 and activation barriers $\Delta E^\#$ are given in the legends. (a) CASSCF calculations on the CF_3CO rigid scan with different active spaces. (b) CASPT2 calculations performed on top of the CASSCF wavefunctions used to obtained the results in (a). (c) Relaxed scan of CF_3CO with the CASSCF(5,5) method. The CASPT2 result was calculated on the CASSCF geometries. Other active spaces failed to converge upon dissociation. d) Rigid scan considering the full molecule CF_3COCl (with the Cl atom kept 10 Å away). The active spaces correspond to those used in the non-adiabatic dynamics.

the quantum yield of the CF_3CO fragment is 0.86 for 280 nm, then 86 % of the molecules should have internal energy below the activation barrier. The activation barrier is then the energy separating the lower 86 % and upper 14 % of the internal energy distribution. Using this rationale, we obtained values of 0.90 eV for the 254 nm wavelength and 0.71 eV for the 280 nm. Computationally, we estimated the barrier to be 0.69 eV based on the XMS-CASPT2 calculations with several active spaces, see the SI for details. The experimentally estimated values are thus reasonably close to the theoretical calculation, with the latter value being almost in quantitative agreement.

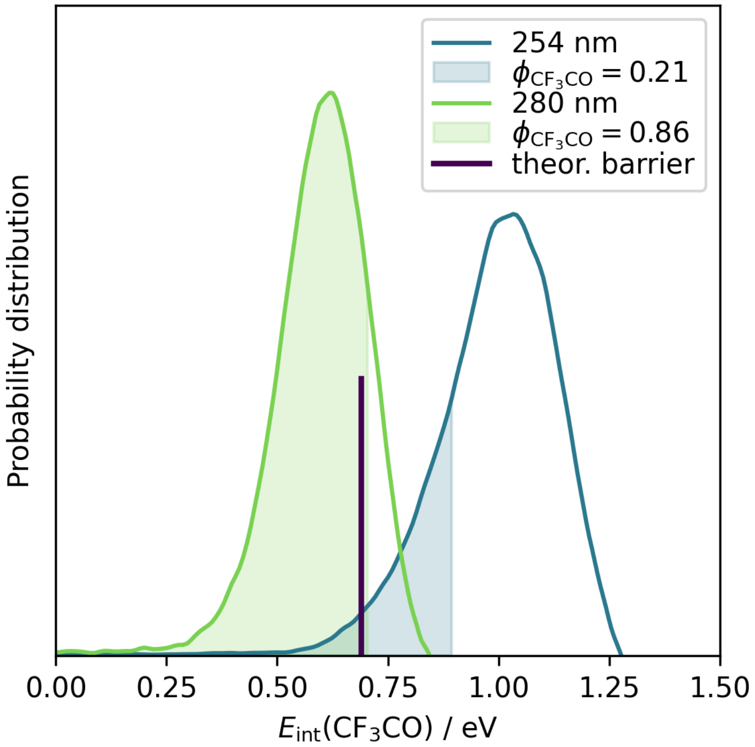

Figure S9: Calculated internal energy distributions in the CF_3CO fragments following the Cl dissociation for 254 and 280 nm. The highlighted areas represent the low-energy parts of the distributions that correspond to the non-dissociating fragments. The edges of the highlighted parts correspond to activation energies for the ground-state dissociation. The theoretically predicted activation barrier is depicted as a black line.

2 Supplementary experimental results

The plots below are shown to support the experimental observations discussed in the article.

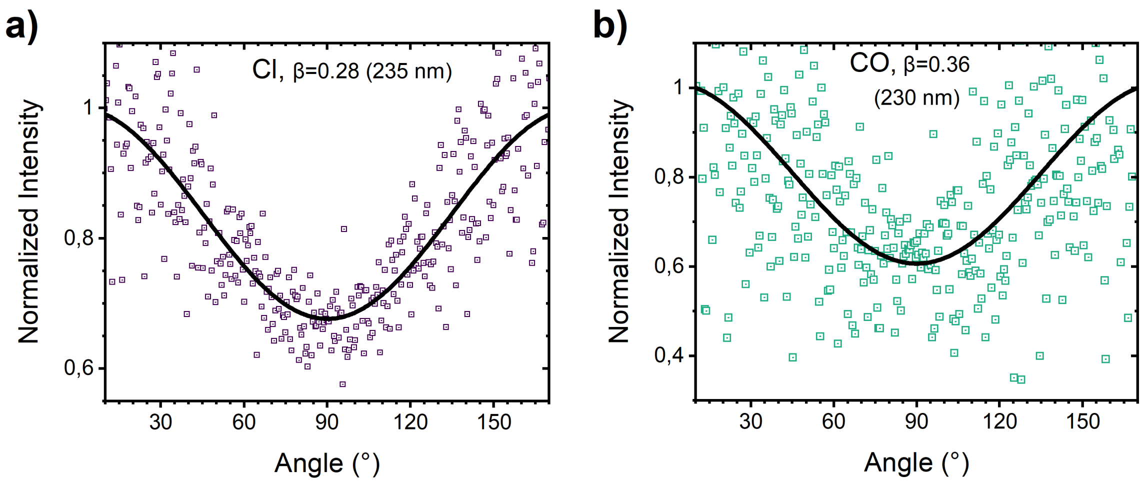

Figure S10: Angular distributions of (a) Cl fragments and (b) CO ($\nu = 0$) fragments acquired after dissociation at 235 nm and 230 nm, respectively.

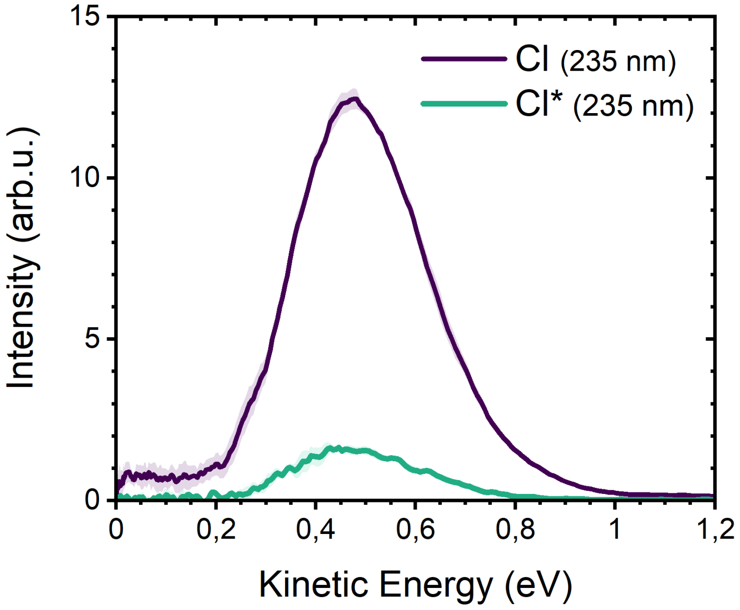

Figure S11: A comparison of the kinetic energy distributions of Cl and Cl* fragments after dissociation at 235 nm.

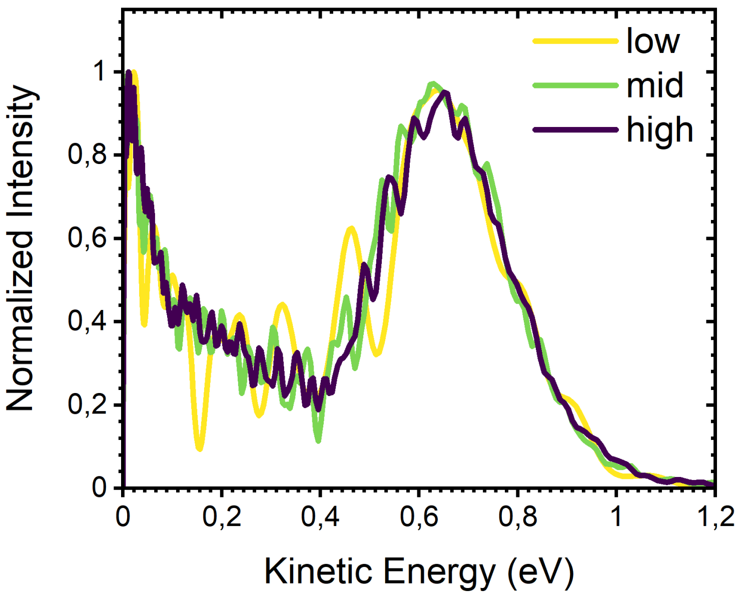

Figure S12: A comparison of the kinetic energy distributions of Cl fragments after dissociation at 193 nm upon different photon flux conditions. The photon flux at the high limit was ≈ 4× higher than in the low photon flux conditions. This measurement indicates no involvement of multiphoton processes.

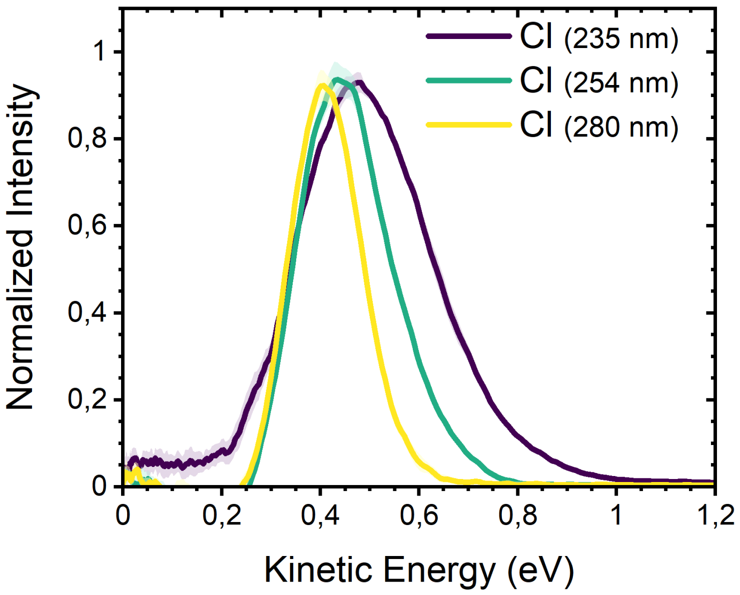

Figure S13: A comparison of the kinetic energy distributions of Cl fragments at different photodissociation wavelengths in the range of the first absorption band. The spectrum indicates a broadening of the distribution together with a small shift of the maxima to higher energies.

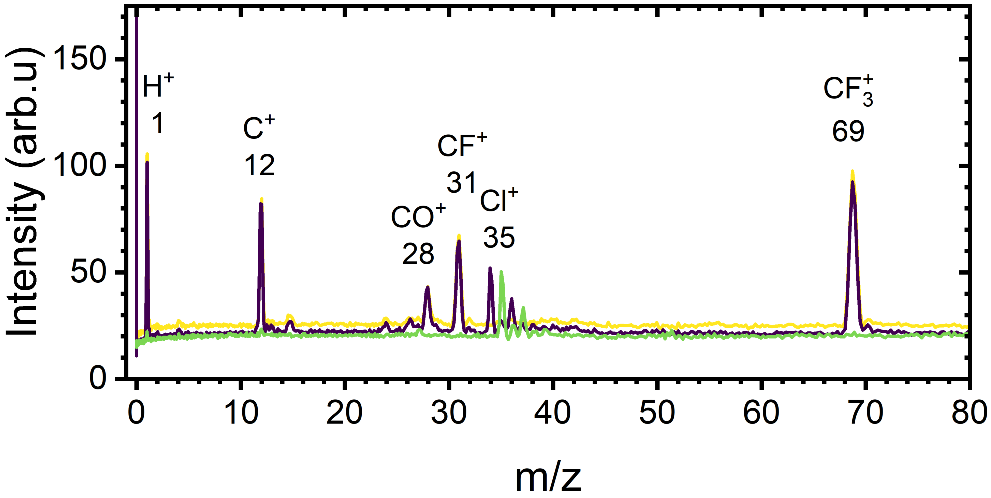

Figure S14: Mass spectrum acquired during the two colour experiment of 193 nm and 235 nm (red), and single color experiments at 193 nm (blue) and 235 nm (green). All the significant peaks are labeled accordingly.

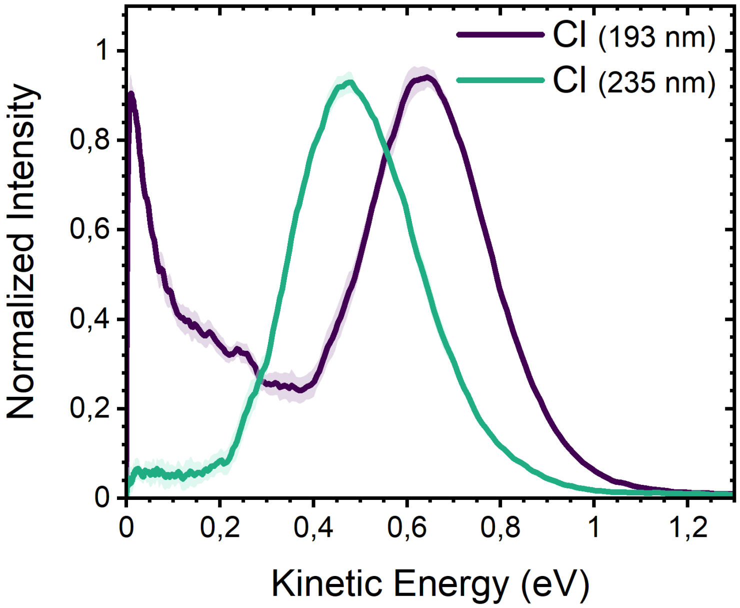

Figure S15: A comparison of the kinetic energy distributions of Cl fragments after photodissociation at 193 nm (green) and 235 nm (red).

References

(S1) Hao, Y.; Liu, L.; Fang, W.-H. Photo-dissociation mechanism of trifluoroacetyl chloride in the gas phase: AIMS dynamic simulations. *The Journal of Chemical Physics* **2021**, *154*, 244303.

(S2) Battaglia, S.; Lindh, R. Extended Dynamically Weighted CASPT2: The Best of Two Worlds. *Journal of Chemical Theory and Computation* **2020**, *16*, 1555–1567.

(S3) Meller, R.; Moortgat, G. K. $CF_3C(O)Cl$: Temperature-dependent (223–298 K) absorption cross-sections and quantum yields at 254 nm. *Journal of Photochemistry and Photobiology A: Chemistry* **1997**, *108*, 105–116.

(S4) McGillen, M. R.; Burkholder, J. B. Gas-phase photodissociation of $CF_3C(O)Cl$ between 193 and 280 nm. *Chemical Physics Letters* **2015**, *639*, 189–194.
